# The Role of Environmental Factors in Lyme Disease Transmission in the European Union: A Systematic Review

**DOI:** 10.3390/tropicalmed9050113

**Published:** 2024-05-15

**Authors:** Christine Giesen, Daniel Cifo, Diana Gomez-Barroso, Rosa M. Estévez-Reboredo, Jordi Figuerola, Zaida Herrador

**Affiliations:** 1 Centro de Salud Internacional Madrid Salud, Ayuntamiento de Madrid, 28006 Madrid, Spain; cgiesen@gmx.net; 2 Escuela Nacional de Sanidad, Instituto de Salud Carlos III, 28029 Madrid, Spain; daniel.cifo@isciii.es; 3 Centro Nacional de Epidemiología, Instituto de Salud Carlos III, 28029 Madrid, Spain; dgomez@isciii.es (D.G.-B.); rm.estevez@isciii.es (R.M.E.-R.); 4 CIBER Epidemiología y Salud Pública (CIBERESP), 28029 Madrid, Spain; jordi@ebd.csic.es; 5 Estación Biológica de Doñana, Consejo Superior de Investigaciones Científicas, 41092 Sevilla, Spain

**Keywords:** climate, *Ixodes*, emerging infectious diseases, Europe, mediterranean, Lyme disease

## Abstract

Background: Lyme disease (LD) is an emergent vector-borne disease caused by *Borrelia* spp. and transmitted through infected ticks, mainly *Ixodes* spp. Our objective was to determine meteorological and environmental factors associated with LD transmission in Europe and the effect of climate change on LD. Materials and methods: A systematic review following the PRISMA guidelines was performed. We selected studies on LD transmission in the European Union (EU) and the European Economic Area (EEA) published between 2000 and 2022. The protocol was registered in the PROSPERO database. Results: We included 81 studies. The impact of environmental, meteorological or climate change factors on tick vectors was studied in 65 papers (80%), and the impact on human LD cases was studied in 16 papers (19%), whereas animal hosts were only addressed in one study (1%). A significant positive relationship was observed between temperature and precipitation and the epidemiology of LD, although contrasting results were found among studies. Other positive factors were humidity and the expansion of anthropized habitats. Conclusions: The epidemiology of LD seems to be related to climatic factors that are changing globally due to ongoing climate change. Unfortunately, the complete zoonotic cycle was not systematically analyzed. It is important to adopt a One Health approach to understand LD epidemiology.

## 1. Introduction

Lyme disease (LD) is a common bacterial vector-borne disease in countries of the European Union (EU) and European Economic Area (EEA). Its pathogenic agent, *Borrelia* sp, is transmitted through the bite of infected ticks, mainly *Ixodes* spp. [1,2,3]. Other ticks that are present in Europe, like *Dermacentor* spp. and *Haemaphysalis* spp., have been identified as carriers of *Borrelia* spp. spirochete, although little is known about their vector competence [4,5,6]. Sporadic infection and transmission by *Rhipicephalus* spp, *Hyalomma* spp. and *Amblyomma* spp. have been reported [7,8]. Deer and rodents are common animal hosts, crucial for the maintenance of the zoonotic cycle in the wild [1,2,3,9].

In the EU/EEA, where it is considered endemic in many places, with more than 360,000 cases reported over the last two decades, the main vector of LD is *Ixodes ricinus* and the main pathogens are *Borrelia afzelii*, *B. garinii* and *B. burgdorferi* [1,2,3]. In 2018, Lyme neuroborreliosis was included on the list of diseases under EU epidemiological surveillance due to increasing trends in the diagnosis of LD cases and vector presence [9,10]. Areas of presence for LD vectors were mapped based on updated information up to March 2022 and include regions in Belgium, Croatia, Czech Republic, France, Germany, Netherlands, Poland and Spain [11].

Environmental factors may influence LD distribution in different ways through their effects on both vector and/or host populations [12,13]. For example, increased temperatures and changes in rainfall patterns may lead to increases in tick populations, through direct and indirect effects on their hosts (e.g., increased rodent populations after mast seeding of trees) [13,14]. However, changes in rainfall can also have negative effects on tick populations, especially if they are registered in arid areas [15]. In addition, *Ixodes ricinus* is expanding into higher altitudes and latitudes as a result of changes in local climate [13], although, depending on the moment when these changes occur, tick populations decrease [14]. Therefore, it is important to understand these relationships and their impact to describe and predict LD dissemination.

This study aimed at reviewing the existing literature to identify the relationship between meteorological/climatic and environmental factors, as well as the influence of climate change, on the presence and/or distribution of vectors and LD circulation in ticks, non-human and human hosts in the EU/EEA.

## 2. Materials and Methods

We conducted a systematic literature review in accordance with PRISMA guidelines [16] to examine the impact of various meteorological and/or environmental factors, as well as their fluctuations, on the presence, dynamics and epidemiology of Lyme disease in the EU/EEA. We registered the study protocol in the PROSPERO database (https://www.crd.york.ac.uk/PROSPERO/) on 11 January 2023, and it was accepted on 22 January 2023 (ID CRD42023391120).

We performed a search in English, French, Portuguese, German, Italian and Spanish and considered only original research studies with quantitative analyses. We regarded proxy metrics (e.g., vector density and abundance) and reservoir populations (comprising animals and humans) as indicative of LD activity. The inclusion and exclusion criteria are shown in Supplementary Materials: Text S1, Table S1. The complete search strategy is detailed in Supplementary Materials: Text S2.

We used a 12-item quality assessment tool based on similar studies and on the Newcastle–Ottawa scale [17,18,19] to evaluate the internal and external validity of the selected publications. Quality was scored as a binary variable (yes/no), and the number of yeses gave the final score: very good (11–12 points), good (9–10 points), moderate (6–8 points), must be improved or unacceptable (<6 points).

The dataset supporting the conclusions of this article is included within the article (and its additional files in Supplementary Materials).

### Study Variables and Data Analysis

We systematically and thematically reviewed the selected papers. We extracted data into evidence tables under different headings, which included identifier, reference, first author, year of publication, journal, vector, disease, host, country of the study, time frame of observed data or year of study, aim, study design, type of environmental and/or meteorological factor according to the definition given by the World Meteorological Organization [20], and data sources, analytical approach, summary of the results, impact on LD incidence (yes/no), projected prevalence, maps (yes/no) and main limitations. To guarantee methodical and uniform data collection, we used a standardized Excel (Version 2010, Microsoft Corporation, Richmond, WA, USA) spreadsheet. References were saved in Zotero software (Version 5.0.67, Corporation for Digital Scholarship, Vienna, VA, USA; www.zotero.org, accessed on 23 January 2023). We used the online tool Rayyan for systematic reviews (Version 2023, Cambridge, MA, USA; https://rayyan.ai/reviews) and Mapchart (Version 2023, https://www.mapchart.net/, accessed on 30 June 2023) and Canva (Version 2023, Sydney, Australia; https://www.canva.com/, accessed on 30 June 2023) for mapping and creating explanatory figures.

## 3. Results

**Figure 1:**
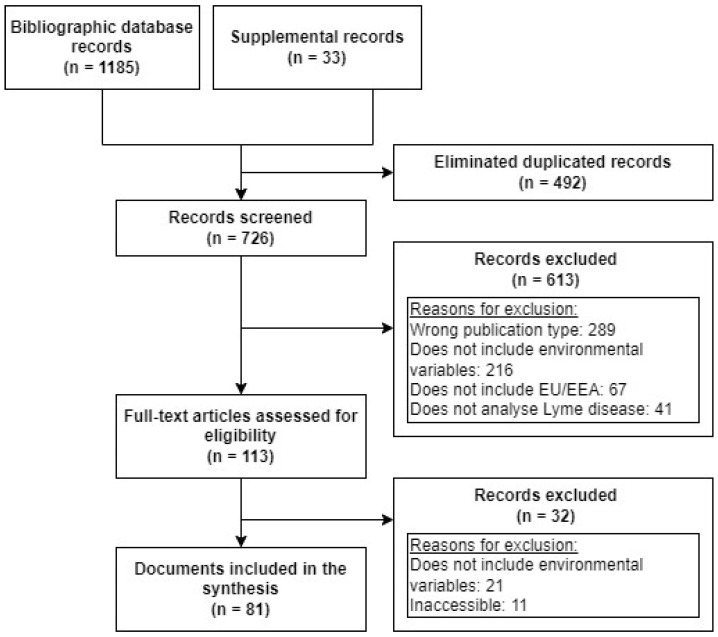
Study selection process.

The systematic search strategy yielded 1218 references. After screening the titles and abstracts, we retained 113 articles for full-text screening. Eighty-one articles met all inclusion criteria (Figure 1).

### 3.1. Descriptive Characteristics of the Studies

**Figure 2:**
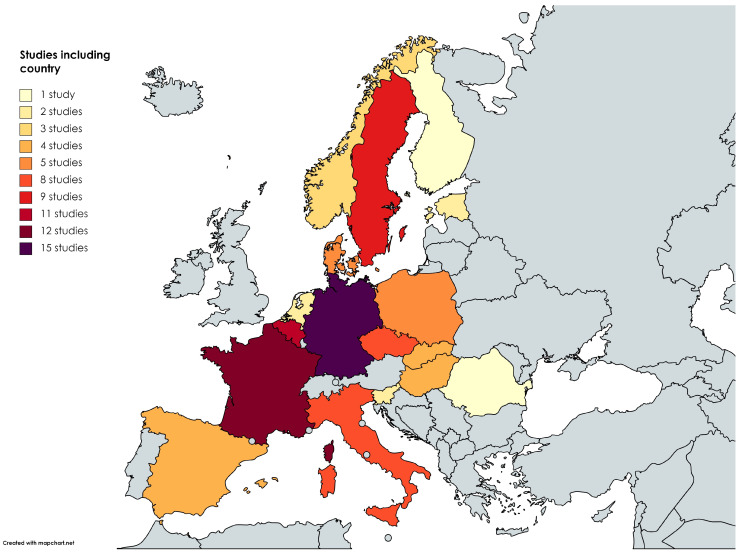
Articles by analyzed countries (n = 81). Countries where no studies were performed are displayed in gray.

Most papers (n = 59) were published between 2011 and 2022. Six studies covered more than one country and three studies focused on the whole European continent (Figure 2). Most research was carried out in Germany (n = 15), France (n = 12) and Belgium (n = 11). The most frequently used types of analyses were association/correlation analyses (n = 56), predictive models (n = 23) and spatial models (n = 15). Twelve studies used two or more different types of modeling approaches (Table 1). Definitions of analyzed variables are specified in Supplementary Materials: Table S2.

Overall, the studies were of medium or good quality (average 11.42 points). The main reasons for scoring lower were the improper identification of the sources for data or the data collection procedures and unclear results (Supplementary Materials: Figure S1).

### 3.2. Lyme Disease Vectors

Sixty-five studies addressed the impact of environmental factors on LD vectors. Twenty-four of these studies additionally analyzed *Borrelia* sp. infection in ticks, which ranged from 0.25% in Germany [60] to 38.0% in Italy [76] (Table 1 and Table 2, Supplementary Materials: Table S3).

Temperature was the most frequently analyzed environmental variable. It was considered a key driver for LD vector abundance [15,28,29,30,31,33,34,37,39,40,41,42,44,45,48,58,60,61,63,64,65,66,73,76,78,81,85,86,88,91,92,94,95], density [36,47,50,62,67,82] and tick bites [80]. Mean [15,29,30,31,33,34,36,38,39,40,44,45,47,48,58,60,61,62,63,64,65,66,67,73,76,80,81,82,85,86,88,92,94,95], maximum [29,42,58,73,78,91] and minimum [29,31,41,50,58,73] daily [29,42,58,61,73,88,91], monthly [15,29,30,31,33,34,36,38,39,40,41,44,45,47,48,50,60,61,63,64,65,66,67,78,81,82,85,86,94,95] or annual [29,39,62,76,92] temperatures were positively related to the abundance and density of *Ixodes ricinus*. Moreover, higher daily near-surface temperatures were associated with earlier tick activities [44,58], and the number of warm days was positively related with the abundance of both larvae [40,66,92] and nymphal stages [40,66,80,86,92]. On the other hand, a negative relationship between temperature and nymph densities and abundance and a positive relationship with adult tick activity were observed [28,50]. In addition, fifty-two studies used temperature variables to model tick abundance and densities [15,29,30,31,33,34,36,38,39,40,41,42,45,47,48,49,50,52,55,56,57,58,60,61,62,63,64,65,66,67,68,73,76,77,78,79,81,82,84,85,86,87,88,89,91,92,93,94,95] and tick bites [80].

Thirty-eight studies analyzed the relationship between humidity and tick abundance [15,22,28,31,33,34,37,42,45,48,49,52,53,58,60,63,64,65,66,68,73,76,77,78,79,81,85,86,88,91,92,95], density [57,62,82,89,97] or tick bites [80]. Daily [42,52,57,58,88] and monthly [15,28,33,34,45,48,53,57,60,63,64,65,85,86] average [15,28,33,34,42,45,48,52,53,57,58,60,63,64,65,85,86,88], maximum [42] and minimum [52] relative humidity were positively related to tick abundance and density. Average daily vapor pressure deficits [42], soil moisture [81], soil pH [48] and soil water capacity [37] were also positively related to tick abundance. The monthly mean relative humidity during spring and autumn and temperature were associated with higher numbers of questing *D. reticulatus* ticks [60]. The degree of ground wetness was also positively related to tick abundance [95]. The numbers of ticks collected were higher when relative humidity was high in the six months preceding tick sampling [15,97]. However, the annual average evapotranspiration showed a negative correlation with *I. ricinus* abundance [92].

Thirty-one studies analyzed precipitation in relation to tick abundance [15,28,29,30,31,34,38,39,40,41,42,45,55,56,64,65,76,77,78,79,84,86,87,91,92,95], density [50,57,67,89] and tick bites of human hosts [80]. Annual [29,87,92], seasonal [29,38,41,80], monthly [29,30,45,65,84] and daily [29,95] mean [29,30,38,41,45,65,80,84,87,92,95], maximum [29,92] and minimum [29,80] precipitation were positively related to tick abundance and tick bites. Mean monthly precipitation was also positively related to *I. ricinus* and *I. persulcatus* abundance [45]. Overall, higher annual precipitation also resulted in higher vector abundance [92], whereas frosty and snowy days resulted in lower tick and larvae abundance [31,92]. However, precipitation from May to September was negatively related to tick abundance [38]. The number of non-rainy days throughout the year was positively related to an increase in human tick bites [80]. Twenty-three studies analyzed and found no relationship between precipitation variables and changes in tick abundance and density [15,28,31,34,39,40,41,42,45,50,55,56,57,67,76,77,78,79,86,87,89,91,95].

Saturation deficit, which describes the functional relationship between saturation vapor pressure, temperature and relative humidity and provides an integrated measure of the drying power of the atmosphere, was analyzed in nine studies in relation to tick abundance and density [48,49,53,62,68,78,86,89,91]. Seven studies observed a positive relationship between *I. ricinus* abundance [48,53,78,86,91] or density [89] and saturation deficit.

Daylight and solar radiation were analyzed in relationship to tick abundance in twelve studies [15,33,40,45,48,65,66,68,81,86,95] and density [36], out of which, five used sun exposure and sunshine duration as model variables [15,48,66,68,95]. Middle infrared reflectance levels [40,45,81], mean monthly solar radiation [36,86] and the sunshine duration [65] and day length [33] were positively associated with tick abundance and density [33,36,40,45,65,81,86]. In addition, daily maximum wind speed was positively related to tick abundance [42].

Fourteen studies analyzed altitude in relationship to tick abundance [15,30,39,40,45,48,72,74,76,87,95] and density [62,82,89]. Altitude, especially at values between 380 and 1400 m, was positively related to tick abundance and density [30,40,48,62,72,74,76,82,89,95].

The enhanced vegetation index (EVI) [39,40,45,76,81] and the normalized difference vegetation index (NDVI) [29,39,40,44,45,76,77,79,80,92,93,94], which are used to quantify vegetation and photosynthetic activity, forest density and extension [15,22,25,28,40,51,54,92] and vegetation period [45,92,98], were used in tick abundance models [15,22,25,28,29,39,40,44,45,51,54,76,77,79,81,92,93,94,98] and in relation to tick bites [80] in twenty studies. Negative correlations were observed between the NDVI and tick abundance in the Czech Republic and Italy [29,76]. However, EVI values [40,81] and vegetation cover [28] were positively related to tick abundance in Norway, Belgium and the Netherlands. Higher tick abundance was observed if the vegetation period started and lasted longer [98]. In contrast, other studies showed that higher values of NDVI correlated positively [39,40,44,77,79,92,94], i.e., densely vegetated and leaved or cultivated areas presented higher tick population abundance.

Thirty-nine papers analyzed tick abundance and density in relation to land uses and land covers [15,22,25,28,29,30,38,40,42,45,48,49,51,52,53,54,55,56,59,62,63,65,66,67,68,71,72,74,79,85,86,87,92,93,95,97,98,100], and one focused on tick bites [80], using data on land coverage [25,28,38,42,45,49,53,54,55,56,65,66,71,72,79,80,92,98,100], forest composition [25,29,30,40,48,49,51,52,55,56,62,65,67,68,74,85,86,87,97,98,100], agricultural land use [40,42,51,54,62,74,79,80,95] and urbanization [22,49,59,67,68,71,79,80] to model tick outcomes. Forests were important drivers for tick abundance and density [25,29,38,42,49,52,53,55,56,62,68,71,72,74,80,85,87,93,95,97,98,100]. Ticks were more present in deciduous [71,72,74,93,97], broad-leaved [42,68,85,97], coniferous [42,62,72], mixed [29,68,93], oak [25,49,74], beech [74,100], pine [74,100] and chestnut [74] forests. The presence of oak trees correlated with a higher abundance of infected nymphs [49]. Higher nymphal infection prevalence was observed in deciduous forests [49], whereas a lower abundance of nymphs occurred in areas with heather [49]. Other forest types and landscapes related to higher tick abundance were hedgerow [52,55,56], woodland [51,52,54], grassland [59,63], spruce [30,97], black locust [74], shrub [25], apple and cherry trees [52], black alder [98] and meadows [66]. In contrast, lower tick numbers were observed in certain types of forests, i.e., coniferous [29,93], broad-leaved [29] and deciduous [29] forests and in older forests [67]. Herb cover [67] and the presence of pole wood [67] also corresponded to a decrease in ticks [67]. Regarding agricultural lands and urbanization, fields and pastures related positively to tick abundance [55,56,59] and parks to tick bites [80]. Moderate forest fragmentation near agricultural areas [15] and an increased forest edge length [25] were also positive predictors. In addition, the farther away any forest road, the higher the abundance of nymphal ticks [49].

Soil-related variables were analyzed in twelve studies in relation to tick abundance [15,29,39,40,48,49,52,55,56,63,74] and tick bites [80], of which, only three studies found positive relationships [49,63,74]. Clay and silt soils were related to a higher abundance of nymphal ticks, and sandy soil was related to higher nymphal infection prevalence [49]. Lower nymphal infection prevalence values, however, were observed with silt soil [49]. Moder humus, i.e., a kind of forest floor in deciduous and mixed-wood forests characterized by a thick layer of fragmented leaves, was strongly associated with nymph abundance in France [48]. Limestone [74] and increased soil water content [63] were also related to increased tick abundance.

Twenty-one studies analyzed animal host abundance in relation to tick abundance and density [22,28,36,37,45,49,51,54,57,61,72,73,74,76,78,81,85,89,92,95,97], which increased with ungulates [97], particularly deer [36,37,45,49,51,74,76,78,81,97], cattle [51,92,95], horse [92,95] or wild boar [49] abundance. Different host species were analyzed in relation with tick abundance, like rodents [49,54,73,97], hares [45,97], shrews [97], birds [97] or foxes [97], of which, roe deer was the most frequently analyzed species [36,37,45,49,51,74,76,81]. No relationships were observed between ticks and moose [45] and mouflon [89] abundance. Landscape connectivity, which is the likelihood that an animal will travel a particular distance through a certain habitat, showed a positive relationship with tick abundance [22].

Human population [38,39,41,45,80,95] and infection [31,36,37,38,81,84] were assessed in relation to tick abundance [31,36,37,38,39,41,45,81,84,95] and tick bites [80]. Human demographic growth, together with sustainable greenhouse gas emissions, were related to increased tick abundance in northern and eastern Europe [41].

Both medium–low- and high-emission scenarios [98] and future climate change projections [41] were positive predictors for current and future *I. ricinus* densities in Scandinavia [98] and Europe [41].

**Figure 3:**
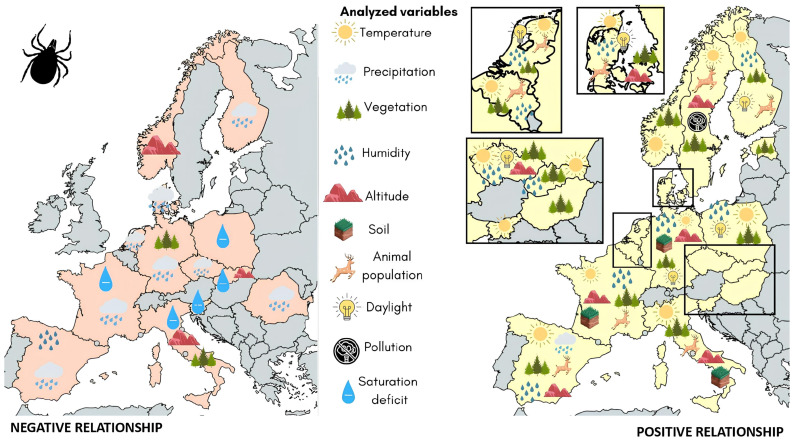
Significant effects of analyzed environmental variables on LD vector abundance and density (n = 65). Countries with significant negative relationships between environmental factors and LD vector abundance and density are shown in orange (**left**), and countries with significant positive relationships between environmental factors and LD vector abundance and density are shown in yellow (**right**). The factors significantly related to vector abundance and density in each country are shown inside the country’s shape. The distribution of environmental variables inside each country’s shape is arbitrary. Countries with no data are shown in gray.

Figure 3 shows the significant effects of the analyzed environmental variables on LD vector abundance and density.

### 3.3. Lyme Disease in Human Hosts

Sixteen studies addressed the impact of environmental variables on human LD cases (Supplementary Materials: Table S4). Two studies focused on specific forms of LD infection, i.e., human neuroborreliosis [99] and erythema migrans (EM), a pathognomonic skin rash that appears following infection in up to 80% of cases [96]. The impact of temperature on human neuroborreliosis [99], EM [96] and LD incidence [24,31,32,69,70] was assessed in seven studies. Mean [31,69,70,96,99] and minimum [31] monthly [31,96,99] and weekly [69,70] air [31,69,70,96,99] and soil [31] temperatures were positive predictors for human LD cases. Higher numbers of winter days with an average temperature below 0 °C in Sweden were related to lower numbers of reported EM cases in the study region [96]. Growing degree days, an indicator of heat accumulation, was also positively related to increased human LD incidence [24]. Only one study showed no relationship between temperature and human LD incidence [32].

Four studies focused on precipitation and human LD incidence. Mean monthly precipitation was positively associated with increases in neuroborreliosis cases [99]. A reduced number of frost days was also positively related to increased human LD incidence because of its critical effect on small mammals, the main hosts for questing larvae and nymphs. This is because of higher host mortality during harsh winters. Ticks are therefore unable to find suitable hosts to survive, which then reflects on lower human LD incidence [31]. However, two other studies found no relationship between precipitation and human LD cases [32,96].

Humidity was assessed in relation to human LD [27,31], neuroborreliosis [99] and EM incident cases [96]. Both the annual cumulative Normalized Difference Water Index (NDWI) [27] and the number of summer days with relative humidity above 86% [96] correlated positively with the number of human cases. In contrast, neither mean monthly relative humidity [96,99] nor soil humidity at the end of winter [31] showed any relation to the number of human cases.

Altitude was a positive predictor for human LD cases [46,90], i.e., human LD cases were also registered at higher altitudes.

Regarding NAO (North Atlantic Oscillation), a cyclical meteorological phenomenon, and human LD incidence, one study showed no relationship [32]. Another study found a negative correlation between NAO index and the number of human cases and was used to accurately predict human cases in Europe [35].

Land use and land cover were assessed in seven studies in relation to human LD [21,26,75,90] and EM incidence [46] and LD seroprevalence [23,83]. Distances to forest [21,23,46,75,83,90], woodland [26,83,90], grassland [26,90], crops and pastures [23,90], urban land [21,90], wetlands [23], moors [26], heathlands [26], meadows [90] and shrubs [90] were used to model the occurrence of human LD cases. The proximity to forests, especially deciduous forests [83], was a positive predictor for LD incidence and prevalence in Belgium [21,23], Italy [75] and Poland [83]. Other factors associated with a higher incidence of human LD cases were distance to semi-natural habitats [23,75], meadows [75] and the distance to small woodlands [90]. However, incidence rates decreased with forest patch density in France [46], and seroprevalence was lower in arable land and grasslands compared to forests and wetlands in Belgium [23].

Three studies analyzed human LD incidence in relation to vegetation. One study found no relationship between the mean monthly NDVI and increases in human LD cases in Slovenia [90], whereas it was a positive predictor for human LD incidence in Belgium [24,26].

Five studies analyzed animal host abundance in relation to LD incidence [21,35,85,90] and prevalence [23] in humans. The presence and abundance of deer [21,23], rodents [35,85,90], birds [90], ungulates [90], carnivores [90], rabbits [90] and wild boars [23] were assessed in Belgium [21,23], Slovenia [90], the Czech Republic [35] and Poland [85]. Roe deer [21] and common vole [35] abundances were related to increased human LD cases in Belgium [21] and the Czech Republic [35].

Human population density [21,26,70,90] and exposure to ticks [21,70,83] were assessed in relation to human LD incidence [21,26,70,90] and prevalence [83] in Belgium [21,26], Slovenia [90], Hungary [70] and Poland [83]. The proportion of people living in spatially dispersed houses and those with higher incomes in periurban areas, as well as high population densities in Belgium [21] and high human outdoor activity in Hungary [70], were positive predictors for human LD incidence.

Climate change predictions, i.e., warmer temperatures, higher CO_2_ emissions and changes in rainfall patterns, among others, showed a positive relationship with human LD incidence in Slovenia as a result of the vector niche shifting to new habitats [90].

**Figure 4:**
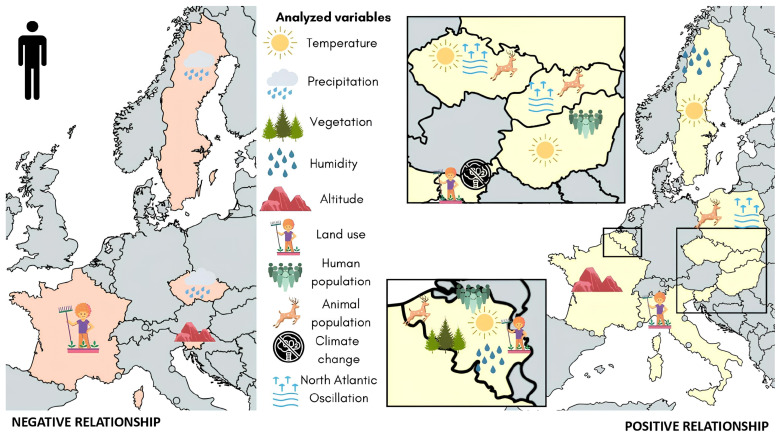
Significant effects of analyzed environmental variables on human LD incidence (n = 16). Countries with significant negative relationships between environmental factors and human LD incidence are shown in orange (**left**) and countries with significant positive relationships between environmental factors and human LD incidence are shown in yellow (**right**). The factors significantly related to vector density in each country are shown inside the country’s shape. The distribution of environmental variables inside each country’s shape is arbitrary. Countries with no data are shown in gray.

Figure 4 shows the significant effects of the analyzed environmental variables on human LD incidence.

### 3.4. Lyme Disease in Animal Hosts

Only one study analyzed animal hosts’ infections in relation to land use. The presence of pastures and natural grasslands in Romania was a positive predictor for *Borrelia* spp. infection in wild boars, roe deer and cattle. Most infections were due to *B. afzelii, B. burgdorferi sensu stricto* and *B. garinii*, although *B. valaisiana, B. spielmanii* and *B. bavariensis* were also detected [59].

### 3.5. Lyme Disease Risk and Expansion

Eight studies analyzed and predicted an expansion of LD to other regions both within countries and cross-border because of the influence of environmental variables [26,41,43,44,58,72,90,98], of which, one study modeled the ecological risk of LD in Europe focusing on the whole transmission cycle under future climate change scenarios. It considered species distribution mapping for animal hosts, i.e., deer, rodents and birds, as well as ticks and human LD risk [43]. Human LD cases will expand to western regions of Slovenia, especially those of lower altitude and rich in wood forests under future climate change scenarios [90], and to southern and northeastern Belgium under the influence of an enhanced NDVI [26]. LD vectors are expected to expand to northern regions of Italy, especially the Piedmont [72], and to northwestern Germany [58] and large regions in Scandinavia as far as 70° N [41,43,44,98]. This expansion will be exacerbated by the presence of coniferous and deciduous forests [72] and black alder trees [98], as well as increases in mean temperatures [41,44,58] and future climate change scenarios [41,43]. According to two studies, by 2030, vectors will have expanded to Nordic countries and central Europe because of increases in temperature and NDVI and future climate change scenarios. However, some models predict that by 2050, LD transmission may be disrupted in some areas of southern Europe because of decreased suitability and no niche overlap between ticks and hosts, due to future predictions of climate change and the transformation of forests into crops [43,44].

## 4. Discussion

Our results show that some studies focused on different *Borrelia* and *Ixodes* species, with the most frequently analyzed being *Borrelia burgdorferi* [21,22,23,24,25,26,27,28,29,30,31,32,35,37,43,45,46,49,50,51,53,56,59,69,70,71,72,74,75,76,79,83,90,92,96,99] and *Ixodes ricinus* [15,22,25,29,30,31,33,34,36,37,38,39,40,41,42,44,45,47,48,49,50,51,52,54,55,56,57,58,59,61,62,63,64,65,66,67,68,71,72,73,74,76,77,78,79,81,82,84,85,86,87,88,89,91,92,93,94,95,97,98,100]. However, new vectors like *Dermacentor reticulatus, Hyalomma lusitanicum, Hyalomma marginatum, Ixodes persulcatus* and *Rhipicephalus sanguineus* have been identified throughout Europe in the last year [11]. Unfortunately, the number of studies analyzing the impact of environment on these other tick species is still reduced.

We observed that different environmental factors, such as temperature, rainfall and different patterns of land use, influence the epidemiology of LD in several countries in the EU/EEA [21,23,25,26,28,29,30,32,34,43,44,45,46,49,50,53,56,60,67,68,69,70,71,72,74,75,76,79,80,82,83,85,88,92,96,97,98,99]. Although these changes affect different elements of the LD zoonotic cycle, most studies focused on the abundance [15,22,25,28,29,30,31,33,34,37,38,39,40,41,42,44,45,48,49,51,52,53,54,55,56,58,59,60,61,63,64,65,66,68,71,72,73,74,76,77,78,79,80,81,84,85,86,87,88,91,92,93,94,95,98,100] and density [36,47,50,57,62,67,82,89,97] of vectors or the incidence on human hosts [21,23,24,26,27,31,32,35,46,69,70,75,83,90,96,99]. To our knowledge, this is the first comprehensive assessment of the impact of these factors on LD expansion in the EU/EEA.

Most of the included papers were published during the second half of the study’s timeframe (2012–2022). This may be due to rising awareness and interest in LD, since the diagnosis of human cases is increasing in the EU/EEA [9,12].

Most studies were carried out in Germany [55,56,57,58,59,60,61,62,63,64,65,66,67,68,79], France [15,46,47,48,49,50,51,52,53,54,55,56] and Belgium [21,22,23,24,25,26,27,28,42,55,56], whereas no or few studies were performed in certain countries where cases of LD or increased presence have been reported, like Baltic countries and Austria [101,102]. Reasons for this may be the differences in reporting and conducting LD surveillance across European countries, the lack and difficulty of diagnosis of LD among clinicians and universal diagnostic guidelines, the usually nonspecific presentation of clinical cases and relatively low awareness among the general population [9,103]. Additionally, only four studies focused on the whole European continent [41,42,43,44]. Therefore, a more comprehensive and cross-border approach is needed to provide the whole picture. While it is interesting to know how LD can spread locally in certain areas or countries, how the disease is expanding throughout the continent also urgently needs to be identified.

We observed contradictory results for some meteorological variables [15,34,37,42,44,45,48,52,58,60,61,63,64,65,81,84,85,86,88,92,95,97]. This might have been a result of different climate zones in Europe, different vegetation cover and different suitability for ticks: southern Europe is characterized by a subtropical climate, where increasing temperatures may even be a limiting factor for suitable tick habitats, whereas higher precipitation may favor tick establishment. However, most parts of Scandinavia present a cold climate, whereas the climate in central Europe is temperate maritime in the west and temperate transitional in the east [104]. Not only the environment, such as certain flora and vegetation cover found in northern latitudes [105], but also fauna, such as *Cervus elaphus* and *Capreolus capreolus*, which are widely present in central and northern Europe and act as hosts for adult ticks, play a major role in LD epidemiology [106]. In addition, some studies analyzed the meteorological or environmental variations within a season, while others focused on several years and analyzed the interannual variations and provided climate change projections. These different methodological approaches may explain some of the observed differences.

Those studies that considered human LD cases mainly focused on LD incidence [21,24,26,27,31,32,35,46,69,70,75,90,96,99], and only two studies analyzed the seroprevalence of LD antibodies in humans [23,83]. This might have been due to the lack of prevalence studies in endemic regions, difficulties in diagnosing LD, the development of new diagnostic methods and the lack of standardized diagnostic protocols and the lack of routine screening for LD antibodies [107,108]. Higher temperature [31,69,70,96,99] and less precipitation [31,99] were associated with an increase in human LD cases. This might be due to human outdoor activity and, thus, exposure to infected ticks, being higher on warm and sunny, non-rainy days, as people might go to the countryside or parks or perform outdoor activities [109]. In addition, the number of questing ticks is higher during summer [110]. Given that extremely high temperatures and droughts were registered during the summer of 2023 in southern and southwestern Europe [111], this may influence future LD epidemiology, leading to higher human LD incidence. In the case of human exposure to LD vectors, additional factors, i.e., animal host abundance or human social behavior, may be important, since the degree of human activity in nature varies and may be affected by the environment. Therefore, both the environment and human behavior have important effects on the whole zoonotic cycle [112].

Most studies focused on tick abundance [15,22,25,28,29,30,31,33,34,37,38,39,40,41,42,44,45,48,49,51,52,53,54,55,56,58,59,60,61,63,64,65,66,68,71,72,73,74,76,77,78,79,80,81,84,85,86,87,88,91,92,93,94,95,98,100] and density [36,47,50,57,62,67,82,89,97] and incidence in human hosts [21,23,24,26,27,31,32,35,46,69,70,75,83,90,96,99], whereas only one study included animal hosts [59]. However, wild animals, i.e., ungulates like deer, are the main hosts of LD, and the spread of the disease and the maintenance of the zoonotic cycle relies completely on them. Some animals nurture ticks and, thus, contribute to the establishment of higher tick populations [113,114], e.g., hundreds of ticks can feed on a single ungulate individual [113]. In addition, some birds and small rodents retain *Borrelia* spp. spirochetes and act as reservoirs for LD [114]. Consequently, changes in the abundance of these vertebrates may have important impacts on the abundance of ticks. This is the case for red deer, where increases in their population may drive important increases in *Ixodes* populations [115]. Therefore, it is necessary to adopt a One Health approach and perform studies that consider all parts of this zoonotic cycle, considering changes in the abundance of hosts but also how environmental conditions affect LD prevalence in their main vertebrate hosts. However, this was only performed in one study [43]. One of the components of this One Health approach is humans themselves, because human behavior when approaching nature or when working in natural environments may have important consequences for exposure to ticks, tick bites and LD [116], and some of these behaviors may be affected by the environment in general and climate in particular. Furthermore, social aspects that influence contact rates between ticks and humans are another important aspect.

The most frequent limitations identified by the studies’ authors were the lack of the analysis of other variables that may influence LD dynamics (n = 44) and concerns about the study and/or model accuracy (n = 11). This might compromise the results of the papers and highlights the importance of developing comprehensive and holistic models when analyzing other variables.

## 5. Limitations

Our study has some shortcomings. We performed a search that was bound to certain inclusion criteria. Other relevant articles might therefore have not been included. However, all included articles were published in English, which is why we believe that most relevant articles were included. We followed the PRISMA guidelines for systematic reviews to limit selection bias. In addition, we observed different methodological qualities in the included studies. Therefore, we used a specific tool to evaluate the studies’ quality, which on average scored very high.

## 6. Conclusions

LD is expanding across Europe. The epidemiology of LD is related to the presence of vectors, which is related to climate and other environmental factors that are changing globally due to ongoing climate change. The environmental factors that most frequently correlated to changes in LD dynamics were temperature, precipitation, humidity and the incursion of human beings into different natural land habitats. Most studies found a positive relationship, although agricultural habitats were associated with decreased human LD incidence. Unfortunately, the complete zoonotic cycle was not systematically analyzed in most papers. Thus, it is difficult to determine the independent impact of environment on the different components of the transmission cycle. It is important to adopt a One Health approach to understand LD epidemiology and to strengthen the surveillance of this emerging disease and its vector. While temperature is increasing worldwide, the impacts of climate change on precipitation present important geographical variations according to the latest Intergovernmental Panel on Climate Change (IPCC) report [117], and consequently, the global impact of climate change on tick populations and LD epidemiology may present important variations within Europe.

## Figures and Tables

**Table 1:** Characteristics of the selected studies (n = 81).

First Author	Year of Publication	Analyzed Countries	Analyzed Vector (Species)	Analyzed Reservoirs and Hosts	*Borrelia* Species	Study Object	Analytical Approach	Scored Points at Quality Assessment
Linard C [21]	2007	Belgium	ND	Humans and animals	*B. burgdorferi* s.l.	Human cases	AM + SM	12
Heylen D [22]	2019	Belgium	*I. ricinus*	Animals	*B. burgdorferi* s.l.	Tick abundance	AM	11
Keukeleire MD [23]	2016	Belgium	ND	Humans and animals	*B. burgdorferi* s.l.	Human cases	AM	12
Barrios JM [24]	2013	Belgium	ND	Humans	*B. burgdorferi* s.l.	Human cases	PM	9
Tack W [25]	2012	Belgium	*I. ricinus*	ND	*B. burgdorferi* s.l.	Tick abundance	AM	12
Barrios JM [26]	2012	Belgium	ND	Humans	*B. burgdorferi* s.l.	Human cases	SM	10
Barrios JM [27]	2012	Belgium	ND	Humans	*B. burgdorferi* s.l.	Human cases	AM	10
Heylen D [28]	2013	Belgium	*I. ricinus*	Animals	*B. burgdorferi* s.l.	Tick abundance	PM	11
Hönig V [29]	2015	Czech Republic	*I. ricinus*	ND	*B. afzelii, B. garinii, B. burgdorferi* s.s., *B. valaisiana, B. lusitaniae, B. spielmanii*	Tick abundance	AM	11
Daniel M [30]	2009	Czech Republic	*I. ricinus*	ND	*B. afzelii*, *B. garinii*, *B. burgdorferi* s.s., *B. valaisiana*	Tick abundance	AM	11
Daniel M [31]	2008	Czech Republic	*I. ricinus*	Humans	*B. burgdorferi* s.l.	Human cases, tick abundance	AM	11
Hubálek Z [32]	2005	Czech Republic	ND	Humans	*B. burgdorferi* s.l.	Human cases	AM	12
Daniel M [33]	2015	Czech Republic	*I. ricinus*	ND	ND	Tick abundance	PM	12
Hubálek Z [34]	2003	Czech Republic	*I. ricinus, H. concinna, D. reticulatus*	ND	ND	Tick abundance	AM	11
Tkadlec E [35]	2019	Czech Republic, Slovakia, Poland	ND	Humans and animals	*B. burgdorferi* s.l.	Human cases	AM	12
Jensen PM [36]	2005	Denmark	*I. ricinus*	Animals	ND	Tick density	AM	11
Jensen PM [37]	2000	Denmark	*I. ricinus*	Animals	*B. burgdorferi* s.l.	Tick abundance	AM	10
Jensen PM [38]	2000	Denmark	*I. ricinus*	ND	ND	Tick abundance	PM	10
Kjær LJ [39]	2019	Denmark, Norway, Sweden	*I. ricinus*	ND	ND	Tick abundance	PM	11
Kjær LJ [40]	2019	Denmark, Norway, Sweden	*I. ricinus*	ND	ND	Tick abundance	PM	12
Porretta D [41]	2013	Europe	*I. ricinus*	ND	ND	Tick abundance	PM + SM	9
Li S [42]	2012	Europe	*I. ricinus*	ND	*B. afzelii, B. garinii*	Tick abundance	PM	12
Li S [43]	2019	Europe	*I. ricinus*	Animals	*B. burgdorferi* s.l.	Human LD risk	PM	10
Fernández-Ruiz N [44]	2020	Europe	*I. ricinus*	ND	ND	Tick abundance	AM	12
Uusitalo R [45]	2022	Finland	*I. ricinus, I. persulcatus*	Animals	*B. burgdorferi* s.l.	Tick abundance	PM	12
Mariet AS [46]	2013	France	ND	Humans	*B. burgdorferi* s.l.	Human cases	AM	11
Vassalo M [47]	2000	France	*I. ricinus*	ND	ND	Tick density	AM	12
Goldstein V [48]	2018	France	*I. ricinus*	ND	ND	Tick abundance	AM	12
Vourc’h G [49]	2016	France	*I. ricinus*	Animals	*B. burgdorferi* s.l.	Tick abundance	AM + SM	12
Paul REL [50]	2016	France	*I. ricinus*	ND	*B. burgdorferi* s.l., *B. miyamotoi*	Tick density	AM	12
Halos L [51]	2010	France	*I. ricinus*	Animals	*B. burgdorferi* s.l.	Tick abundance	AM	12
Wongnak P [15]	2022	France	*I. ricinus*	ND	ND	Tick abundance	AM	12
Boyard C [52]	2007	France	*I. ricinus*	ND	ND	Tick abundance	PM	12
Bourdin A [53]	2022	France	*I. ricinus*	ND	*B. afzelii, B. burgdorferi* s.l., *B. burgdorferi* s.s., *B. garinii, B. lusitaniae, B. valaisiana*	Tick abundance	AM	12
Perez G [54]	2016	France	*I. ricinus*	Animals	ND	Tick abundance	PM	12
Ehrmann S [55]	2017	France, Belgium, Germany, Sweden, Estonia	*I. ricinus*	ND	ND	Tick abundance	AM	10
Ehrmann S [56]	2018	France, Belgium, Germany, Sweden, Estonia	*I. ricinus*	ND	*B. burgdorferi* s.l.	Tick abundance	PM	10
Brugger K [57]	2018	Germany	*I. ricinus*	Animals	ND	Tick density	PM	9
Nolzen H [58]	2022	Germany	*I. ricinus*	ND	ND	Tick abundance	PM + SM	11
Răileanu C [59]	2021	Germany	*I. ricinus*	Humans and animals	*B. afzelii*, *B. burgdorferi* s.s., *B. garinii*, *B. valaisiana*, *B. spielmanii*, *B. bavariensis*	Tick abundance, tick and host infection	AM	12
Kohn M [60]	2019	Germany	*D. reticulatus*	ND	*B. miyamotoi, B. afzelii*	Tick abundance	AM	11
Brugger K [61]	2017	Germany	*I. ricinus*	Animals	ND	Tick abundance	AM	12
Boehnke D [62]	2015	Germany	*I. ricinus*	ND	ND	Tick density	SM	12
Schwarz A [63]	2009	Germany	*I. ricinus*	ND	ND	Tick abundance	AM + SM	12
Vollack K [64]	2017	Germany	*I. ricinus*	ND	ND	Tick abundance	AM	12
Schulz M [65]	2014	Germany	*I. ricinus*	ND	ND	Tick abundance	AM	12
Gethmann J [66]	2020	Germany	*I. ricinus*	ND	ND	Tick abundance	AM	12
Lauterbach R [67]	2013	Germany	*I. ricinus*	ND	ND	Tick density	PM	12
Hauck D [68]	2020	Germany	*I. ricinus, I. inopinatus, I. frontalis, I. hexagonus*	ND	ND	Tick abundance	AM	12
Trájer A [69]	2013	Hungary	ND	Humans	*B. burgdorferi* s.l.	Human cases	AM	12
Trájer A [70]	2014	Hungary	ND	Humans	*B. burgdorferi* s.l.	Human cases	PM	11
Hornok S [71]	2017	Hungary	*I. ricinus, D. reticulatus, D. marginatus, H. inermis, H. concinna*	ND	*B. burgdorferi* s.l.	Tick abundance	AM	12
Garcia-Vozmediano A [72]	2020	Italy	*I. ricinus, D. marginatus*	Animals	*B. burgdorferi* s.l., *B. miyamotoi*	Tick abundance	AM	9
Rosà R [73]	2007	Italy	*I. ricinus*	Animals	ND	Tick abundance	PM	12
Rizzoli A [74]	2002	Italy	*I. ricinus*	Animals	*B. burgdorferi* s.l.	Tick abundance	PM + SM	12
Zanzani SA [75]	2019	Italy	ND	Humans	*B. burgdorferi* s.l.	Human cases	SM	12
Altobelli A [76]	2008	Italy	*I. ricinus*	Animals	*B. burgdorferi* s.l.	Tick abundance	AM + SM	11
Bisanzio D [77]	2008	Italy	*I. ricinus*	ND	ND	Tick abundance	AM	12
Tagliapietra V [78]	2011	Italy	*I. ricinus*	Animals	ND	Tick abundance	AM	12
Rosà R [79]	2018	Italy, Germany, Czech Republic, Slovakia, Hungary	*I. ricinus*	ND	*B. burgdorferi* s.l.	Tick abundance	AM	12
Garcia-Martí I [80]	2017	Netherlands	*I. ricinus*	ND	ND	Tick bites	AM + SM	12
Swart A [81]	2014	Netherlands	*I. ricinus*	Animals	ND	Tick abundance	PM + SM	10
Qviller L [82]	2014	Norway	*I. ricinus*	ND	ND	Tick density	AM	12
Kiewra D [83]	2018	Poland	ND	Humans	*B. burgdorferi* s.l.	Human cases	AM	12
Buczek A [84]	2014	Poland	*I. ricinus*	ND	ND	Tick abundance	AM	12
Dyczko D [85]	2022	Poland	*I. ricinus*	Animals	*B. afzelii, B. garinii, B. valaisiana, B. lusitaniae, B. miyamotoi*	Tick abundance	AM	12
Kiewra D [86]	2014	Poland	*I. ricinus*	ND	ND	Tick abundance	AM	12
Domşa C [87]	2018	Romania	*I. ricinus*	ND	ND	Tick abundance	PM	12
Pangrácová L [88]	2013	Slovakia	*I. ricinus*	ND	ND	Tick abundance	AM	11
Kazimírová M [89]	2016	Slovakia	*I. ricinus*	Animals	ND	Tick density	AM	12
Donša D [90]	2021	Slovenia	ND	Humans and animals	*B. burgdorferi* s.l.	Human cases	PM + SM	12
Knap N [91]	2009	Slovenia	*I. ricinus*	ND	ND	Tick abundance	AM	12
Ruiz-Fons F [92]	2012	Spain	*I. ricinus*	Animals	*B. burgdorferi* s.l.	Tick abundance	AM + SM	12
Estrada-Peña A [93]	2001	Spain	*I. ricinus*	ND	ND	Tick abundance	AM	10
Alonso-Carné J [94]	2016	Spain	*I. ricinus*	ND	ND	Tick abundance	AM	11
Barandika JF [95]	2006	Spain	*I. ricinus, H. punctata*	Animals	ND	Tick abundance	AM	12
Bennet L [96]	2006	Sweden	ND	Humans	*B. burgdorferi* s.l.	Human cases	AM	12
Jaenson TG [97]	2009	Sweden	*I. ricinus*	Animals	ND	Tick density	AM	10
Jaenson TG [98]	2011	Sweden	*I. ricinus*	ND	ND	Tick abundance	AM + SM + PM	12
Keith K [99]	2022	Sweden	ND	Humans	*B. burgdorferi* s.l.	Human cases	AM	12
Lindström A [100]	2003	Sweden	*I. ricinus*	ND	ND	Tick abundance	AM	10

AM: association/correlation models; LD: Lyme disease; ND: no data; PM: predictive model; SM: spatial model. Study quality: the quality of the included studies was assessed. Further information can be found in the Material and Methods and Supplementary Materials: Figure S1.

**Table 2:** Observed *Borrelia* and *Ixodes* species in different countries.

Analyzed Species	Countries
Analyzed *Borrelia* species	
*B. afzelii*	Czech Republic [29,30], Germany [59,60], Poland [85]
*B. bavariensis*	Germany [59]
*B. burgdorferi* s.l.	Belgium [21,22,23,24,25,26,27,28,56], Czech Republic [31,32,35,79], Denmark [37], Estonia [56], Finland [45], France [46,49,50,51,53,56], Germany [56,79], Hungary [69,70,71,79], Italy [72,74,75,76,79], Poland [35,83], Slovakia [35,79], Slovenia [90], Spain [92], Sweden [56,96,99]
*B. burgdorferi* s.s.	Czech Republic [29,30], France [53], Germany [59]
*B. garinii*	Czech Republic [29,30], Germany [59], Poland [85]
*B. lusitaniae*	Czech Republic [29], Poland [85]
*B. miyamotoi*	France [50], Germany [60], Italy [72], Poland [85]
*B. spielmanii*	Czech Republic [29], Germany [59]
*B. valaisiana*	Czech Republic [29,30], Germany [59], Poland [85]
Analyzed vector species	
*D. marginatus*	Hungary [71], Italy [72]
*D. reticulatus*	Czech Republic [34], Germany [60], Hungary [71]
*H. concinna*	Czech Republic [34], Hungary [71]
*H. inermis*	Hungary [71]
*H. punctata*	Spain [95]
*I. frontalis*	Germany [68]
*I. hexagonus*	Germany [68]
*I. inopinatus*	Germany [68]
*I. persulcatus*	Finland [45]
*I. ricinus*	Belgium [22,25,28,55,56], Czech Republic [29,30,31,33,34,79], Denmark [36,37,38,39,40], Estonia [55,56], Finland [45], France [15,47,48,49,50,51,52,53,54,55,56], Germany [55,56,57,58,59,61,62,63,64,65,66,67,68,79], Hungary [71,79], Italy [72,73,74,76,77,78,79], Netherlands [80,81], Norway [39,40,82], Poland [84,85,86], Romania [87], Slovakia [79,88,89], Slovenia [91], Spain [92,93,94,95], Sweden [39,40,55,56,97,98,100]

## Data Availability

The dataset supporting the conclusions of this article is included within the article (and its additional files in the Supplementary Materials).

## Supplementary Materials

The following supporting information can be downloaded at https://www.mdpi.com/article/10.3390/tropicalmed9050113/s1: Text S1. Inclusion and exclusion criteria. Text S2. Search strategy. Figure S1. Scored points in quality assessment (n = 81). Table S1. Eligibility criteria. Table S2. Definition of analyzed variables. Table S3. Effect of different environmental variables on vectors. Table S4. Effect of different environmental variables on LD in human hosts.
