# Supplementary material for: The Role of Environmental Factors in Lyme Disease Transmission in the European Union: A Systematic Review
Source: Trop Med Infect Dis. 2024 May 15;9(5):113. doi: 10.3390/tropicalmed9050113 (PMC11125681; doi:10.3390/tropicalmed9050113)
Supplement: Supplementary file 1 [file tropicalmed-09-00113-s001.zip › tropicalmed-2864458-supplementary.pdf]

Text S1: Inclusion and Exclusion Criteria

All articles that addressed the presence of LD and evaluated the impact of environmental factors in LD in the EU/EEA were included. We included both EU and EEA countries because they share common meteorological and environmental features and report surveillance outcomes to the ECDC. The PRISMA guideline steps were followed, specifying the reasons for exclusion [16]. A registry was kept, identifying each study as included or excluded and the exclusion reason. Duplicates were removed. We only included original research studies. Studies that did not assess the impact of environmental factors on LD in humans, hosts or its vectors or were carried out outside the EU/EEA were excluded (supplementary Table 1).

The recovered abstracts were read by two researchers independently (double peer review), and inclusion/exclusion criteria were applied to produce the final list of publications to be read in full text. During the second filtering, those articles that did not fit the inclusion criteria were also excluded. If there were uncertainties whether to include a study, a third reviewer assessed if the article should be included or not. To assess the inclusion or exclusion the following questions were answered as yes, no or uncertain:

1. Does the study address the study area?
2. Does the study address LD?
3. Does the study consider meteorological, environmental or climate change factors?
4. Does the study assess the relationship between LD and meteorological, environmental or climate change factors?

The reference was discarded when the answer to any question was "no".

Text S2: Search Strategy

Two main reviewers searched for indexed articles published in the PubMed, Scopus, Embase and CENTRAL databases and published between 01/01/2000 and 31/12/2022. The search was performed in Spanish, English, French, Italian, German and Portuguese.

We also cross-checked the reference list of all included articles for relevant studies.

Our study area was the EU/EEA. Articles needed to refer to the presence of LD and/or its vectors and present data regarding meteorological, environmental or climate change variables (from this point on, environmental factors). All databases were searched for original articles.

The following search terms were screened in title, abstract and keywords using the AND Boolean logic operator and using the option 'title/abstract':

1. (Lyme OR lyme borreliosis OR Borrelia burgdorferi OR Borrelia burgdorferi infection OR lyme infection OR Borrelia mayonii OR Borrelia afzelii OR Borrelia garinii OR Borrelia bavariensis OR Borrelia spielmanii OR Borrelia lusitaniae OR Borrelia mayonii infection OR Borrelia afzelii infection OR Borrelia garinii infection OR Borrelia bavariensis infection OR Borrelia spielmanii infection OR Borrelia lusitaniae infection OR borreliosis OR borreliosis infection OR neuroborreliosis) AND

2. (climat* OR environment* OR temperature OR warm* OR meteo* OR rainfall OR humidity OR altitude OR drought OR flood OR habitat OR landscape use OR land use OR climate change OR meteorological) AND
3. (Austria OR Belgium OR Bulgaria OR Croatia OR Cyprus OR Czech Republic OR Denmark OR Estonia OR Finland OR France OR Germany OR Greece OR Hungary OR Iceland OR Ireland OR Italy OR Latvia OR Liechtenstein OR Lithuania OR Luxembourg OR Malta OR Netherlands OR Norway OR Poland OR Portugal OR Romania OR Slovakia OR Slovenia OR Spain OR Sweden OR Europe OR European Union).

Supplementary figures

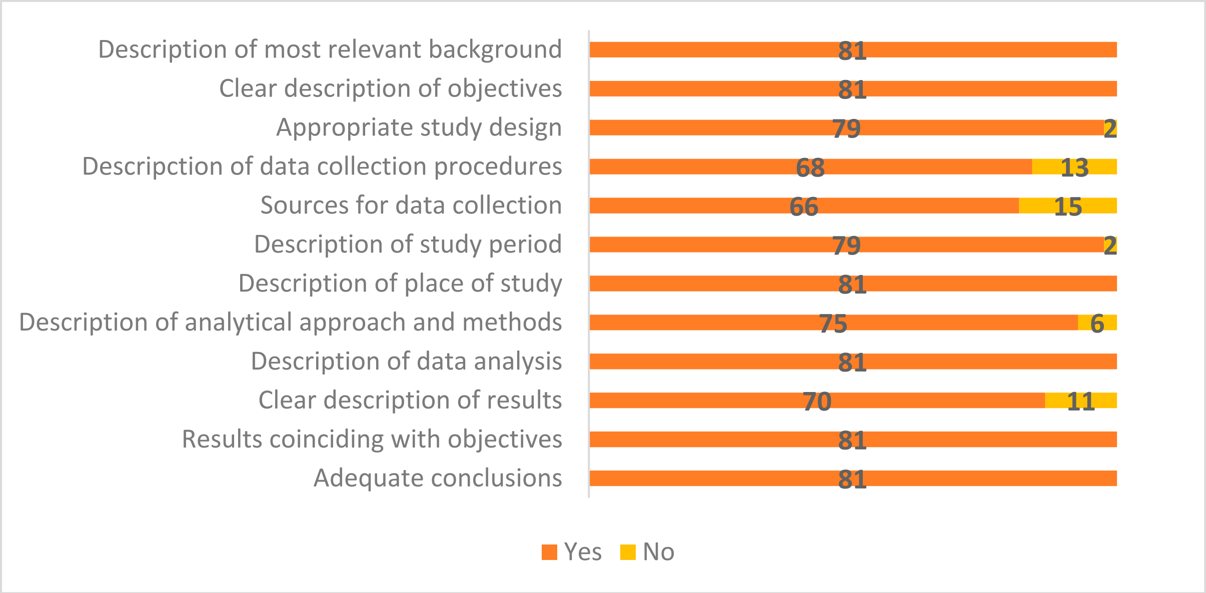

Figure S1. Scored points in quality assessment (n=81).

Supplementary tables

Table S1. Eligibility criteria

Inclusion criteria	Exclusion criteria
Original research studies	Other type of study
Studies must refer to LD in humans, hosts or its vectors and analyze the impact of environmental and meteorological factors or climate change	Not assessing the impact of environmental and meteorological factors or climate change on LD
Published between 01/01/2000 and 31/12/2022	Published before 01/01/2000 or after 31/12/2022

Languages: English, French, Portuguese, Italian, German and Spanish	Not addressing any of the included countries or regions

Table S2. Definition of analyzed variables.

**Variable**	**Definition**
Meteorological	
Temperature	A physical quantity that quantitatively expresses the perceptions of hotness and coldness.
Precipitation	Any liquid or frozen water that forms in the atmosphere and falls back to Earth.
Humidity	The amount of water vapor in the air.
NDWI	A remote sensing-derived index related to liquid water.
Wind	The movement of air caused by the uneven heating of the Earth by the sun and the Earth's own rotation.
NAO	A cyclical meteorological phenomenon over the North Atlantic Ocean of fluctuations in the difference of atmospheric pressure at sea level between the Icelandic Low and the Azores High.
Daylight	The natural light of the day.
Altitude/Elevation	The height of a point in relation to sea level or ground level.
Saturation deficit	Index which describes the functional relationship between saturation vapour pressure, temperature and relative humidity and thus provides an integrated measure of the drying power of the atmosphere.
Soil	The upper layer of earth in which plants grow, typically consisting of a mixture of organic remains, clay and rock particles.
Environmental	
Land use	How people are using the land.
Land cover	The physical land type such as forest or open water.
Vegetation	Plants considered collectively, especially those found in a particular area or habitat.
EVI	Index which is used to quantify vegetation, forest density and extension.
NDVI	Index which is used to quantify vegetation, forest density and extension.
Climate change	
Climate change	Long-term shifts in temperatures and weather patterns.
Emissions	The production and discharge of gas or radiation.

Pollution	The presence in or introduction into the environment of a substance which has harmful or poisonous effects.
Disease	
Human population density & exposure	A measurement of human population per unit land area.
Human LD infection	Confirmed human LD cases.
Animal host population density	A measurement of animal host population per unit land area.
Vector population density	A measurement of vector population per unit land area.

EVI: enhanced vegetation index; LD: Lyme disease; NAO: North Atlantic Oscillation; NDVI: normalized difference vegetation index; NDWI: normalized difference water index. Source: World Meteorological Organization (2023) [117].

Table S3. Effect of different environmental variables on vectors

Variable	Country/Region	Vector density	Vector expansion	Other: tick bites, vector infection	Vector species
**Temperature**	Belgium [27,41,54,55], Czech Republic [28–30,32,33,78], Denmark [35,37–39], Estonia [54,55], Europe [40,43], Finland [44], France [15,46–49,51,54,55], Germany [54–57,59–67,78], Hungary [78], Italy [72,75–78], Netherlands [79,80], Norway [38,39,81], Poland [83–85], Romania [86], Slovakia [78,87,88], Slovenia [90], Spain [91–94], Sweden [38,39,54,55]	Increase [15,27–30,32,33,35,37–41,43,44,46,47,49,57,59–66,72,75,77,80,81,84,85,87,90,91,93,94]	Increase [40,43,57]	Increased tick bites [79]	*D. reticulatus* [33,59], *H. concinna* [33], *H. punctata* [94], *I. frontalis* [67], *I. hexagonus* [67], *I. inopinatus* [67], *I. persulcatus* [44], *I. ricinus* [15,27–30,32,33,35,37–41,43,44,46–49,51,54–57,60–67,72,75–78,80,81,83–88,90–94], tick bites [79]
**Precipitation**	Belgium [27,41,54,55], Czech Republic [28–30,33,78], Denmark [37–39], Estonia [54,55], Europe [40], Finland [44], France [15,49,54,55], Germany [54–56,63,64,66,78], Hungary [78], Italy [75–78], Netherlands [79], Norway [38,78], Poland [85], Romania	Increase [40,91]; Decrease [28–30,37,44,49,63,64,79,86,91,94]	Increase [40]	Decreased tick bites [79]	*D. reticulatus* [33], *H. concinna* [33], *H. punctata* [94], *I. persulcatus* [44], *I. ricinus* [15,27–30,33,37–41,44,49,54–56,63,64,66,75–78,85,86,88,90,91,94], tick bites [79]

	[86], Slovakia [78,88], Slovenia [90], Spain [91,94], Sweden [38,39,54,55]				
**Humidity**	Belgium [21,27,41], Czech Republic [30,32,33,78], Denmark [36], Finland [44], France [15,47,48,51,52], Germany [57,59–65,67,78], Hungary [78], Italy [72,75–78], Netherlands [79,80], Norway [81], Poland [83–85], Slovakia [78,87,88], Slovenia [90], Spain [91,94], Sweden [96]	Decrease [91]; Increase [15,27,32,33,36,41,44,47,51,52,57,59,60,62–64,80,83–85,87,94,96]	Increase [57]	Not assessed	*D. reticulatus* [33,59], *H. concinna* [33], *H. punctata* [94], *I. frontalis* [67], *I. hexagonus* [67], *I. inopinatus* [67], *I. persulcatus* [44], *I. ricinus* [15,21,27,30,32,33,36,41,44,47,48,51,52,57,60–65,67,75–78,80,81,83–85,87,88,90,91,94,96], tick bites [79]
**Wind**	Belgium [41], Czech Republic [33], Germany [65], Poland [85]	Increase [41]	Not assessed	Not assessed	*D. reticulatus* [33], *H. concinna* [33], *I. ricinus* [33,41,65,85]
**Saturation deficit**	France [47,48,52], Germany [61,67], Italy [77], Poland [85], Slovakia [88], Slovenia [90]	Decrease [47,52,77,85,88,90]	Not assessed	Not assessed	*I. frontalis* [67], *I. hexagonus* [67], *I. inopinatus* [67], *I. ricinus* [47,48,52,61,67,77,85,88,90]
**Land use and land cover**	Belgium [21,24,27,41,54,55], Czech Republic [28,29,78], Denmark [37,39], Estonia [54,55], Finland [44], France [47,48,50–55], Germany [54,55,58,61,62,64–67,78], Hungary [70,78], Italy [71,73,78], Netherlands [79], Norway [39], Poland [84,85], Romania [86], Slovakia [78], Spain [91,92,94], Sweden [39,54,55,96,97,99]	Increase [24,28,29,37,41,48,50–55,58,61,62,65,67,70,71,73,84,86,92,94,96,97,99]; Decrease [28,66,92]	Increase [71]	Increased tick bites [79], increased vector infection [58]	*D. marginatus* [70,71], *D. reticulatus* [70], *H. concinna* [70], *H. inermis* [70], *H. punctata* [94], *I. frontalis* [67], *I. hexagonus* [67], *I. inopinatus* [67], *I. persulcatus* [44], *I. ricinus* [21,24,27–29,37,39,41,44,47,48,50–55,58,61,62,64–67,70,71,73,78,84–86,91,92,94,96,97,99], tick bites [79]
**Vegetation and NDVI**	Belgium [21,24,27], Czech Republic [28,78], Denmark [38,39], Europe [43], Finland [44], France [15,50,53], Germany [78], Hungary [78], Italy [75,76,78], Netherlands [79,80], Norway [38,39],	Increase [15,24,27,38,39,43,76,78,80,91,93,97]; Decrease [28,75]	Not assessed	Not assessed	*I. persulcatus* [44], *I. ricinus* [15,21,24,27,28,38,39,43,44,50,53,75,76,78,80,91–93,97], tick bites [79]

	Slovakia [78], Spain [91–93], Sweden [38,39,97]				
**Soil**	Belgium [54,55], Czech Republic [28], Denmark [38,39], Estonia [54,55], Finland [44], France [15,47,48,51,54,55], Germany [54,55,62], Italy [73], Netherlands [79], Norway [38,39], Sweden [38,39,54,55]	Increase [47,48,62,73]	Not assessed	Not assessed	*I. persulcatus* [44], *I. ricinus* [15,28,38,39,47,48,51,54,55,62,73], tick bites [79]
**Daylight**	Czech Republic [32], Denmark [35,39], Finland [44], France [15,47], Germany [64,65,67], Netherlands [80], Norway [39], Poland [85], Spain [94], Sweden [39]	Increase [32,35,39,44,64,80,85]	Not assessed	Not assessed	*H. punctata* [94], *I. frontalis* [67], *I. hexagonus* [67], *I. inopinatus* [67], *I. persulcatus* [44], *I. ricinus* [15,32,35,39,44,47,64,65,80,85,94]
**Altitude/Elevation**	Czech Republic [29], Denmark [38,39], Finland [44], France [15,47], Germany [61], Italy [71,73,75], Norway [38,39,81], Romania [86], Slovakia [88], Spain [94], Sweden [38,39]	Decrease [73,81,88]; Increase [29,39,47,61,71,75,94]	Increase [71]	Not assessed	*D. marginatus* [71], *H. punctata* [94], *I. persulcatus* [44], *I. ricinus* [15,29,38,39,44,47,61,71,73,75,78,81,86,88,94]
**Human population density and exposure**	Denmark [37], Europe [40], Finland [44], Netherlands [79], Norway [38], Spain [94], Sweden [38]	Increase [40,79]	Not assessed	Increased tick bite [79]	*I. persulcatus* [44], *I. ricinus* [37,38,40,44,94], tick bites [79]
**Human LD infection**	Czech Republic [30], Denmark [35–37], Netherlands [80], Poland [83]	Increase [36]	Not assessed	Not assessed	*I. ricinus* [30,35–37,80,83]
**Animal host population density**	Belgium [21,27], Denmark [35,36], Finland [44], France [48,50,53], Germany [56,60], Italy [71–73,75,77], Netherlands [80], Slovakia [88], Spain [91,94], Sweden [96]	Increase [21,35,36,44,48,50,53,71–73,75,77,80,91,94,96]	Not assessed	Not assessed	*D. marginatus* [71], *H. punctata* [94], *I. persulcatus* [44], *I. ricinus* [21,27,35,36,44,48,50,53,56,60,71–73,75,77,80,88,91,94,96]
**Others**	Europe [40], Poland [83], Sweden [97]	Increase [40,97]	Increase [40,97]	Not assessed	*I. ricinus* [40,83,97]

NDVI: normalized difference vegetation index.

Table S4. Effect of different environmental variables on LD in human hosts

Variable	Country/Region	Effect on LD incidence	Effect on LD prevalence	Effect on LD expansion	LD measure (Case/Seroprevalence)
**Temperature**	Belgium [23], Czech Republic [30,31], Hungary [68,69], Sweden [95,98]	Increase [23,30,68,69,95,98]	Not assessed	Not assessed	Human EM cases [95], human LD cases [23,30,31,69], human LB & EM cases [68], human neuroborreliosis cases [98]
**Precipitation**	Czech Republic [30,31], Sweden [95,98]	Decrease [30,98]	Not assessed	Not assessed	Human EM cases [95], human LD cases [30,31], human neuroborreliosis cases [98]
**Vegetation/NDVI**	Belgium [23,25], Slovenia [89]	Increase [23,25]	Not assessed	Increase [25]	Human LD cases [23,25,89]
**Land use and land cover**	Belgium [20,22,25], France [45], Italy [74], Poland [82], Slovenia [89]	Increase [20,74,89]; Decrease [45]	Increase [22,82]; Decrease [22]	Increase [89]	Human EM cases [45], human LD cases [20,25,74,89], human LD seroprevalence [22,82]
**Humidity and NDWI**	Belgium [26], Czech Republic [30], Sweden [95,98]	Increase [26,95]	Not assessed	Not assessed	Human EM cases [95], human LD cases [26,30], human neuroborreliosis cases [98]
**Altitude/Elevation**	France [45], Slovenia [89]	Increase [45]; Decrease [89]	Not assessed	Not assessed	Human EM cases [45], human LD cases [89]
**Human population density and exposure**	Belgium [20,25], Hungary [69], Poland [82], Slovenia [89]	Increase [20,25,69]	Not assessed	Increase [25,89]	Human LD cases [20,25,69,89], human LD seroprevalence [82]
**Animal host population density**	Belgium [20,22], Czech Republic [34], Poland [34], Slovakia [34], Slovenia [89]	Increase [20,34]	Not assessed	Increase [89]	Human LD cases [20,34,89], human LD seroprevalence [22]
**Vector population density**	Czech Republic [30], Slovenia [89]	Not assessed	Not assessed	Not assessed	Human LD cases [30,89]
**Other**	Czech Republic [31,34], Poland [34], Slovakia [34], Slovenia [89]	Increase [34,89]	Not assessed	Increase [89]	Human LD cases [31,34,89]

EM: erythema migrans; LD: Lyme disease; NAO: North Atlantic Oscillation; NDVI: normalized difference vegetation index; NDWI: normalized difference water index.

References

1. CDC Lyme Disease. Available online: https://www.cdc.gov/lyme/index.html (accessed on 17 December 2022).
2. World Health Organization Regional Office for Europe/European Centres for Disease Control: Lyme Borreliosis in Europe. Available online:

https://www.ecdc.europa.eu/sites/default/files/media/en/healthtopics/vectors/world-health-day-2014/Documents/factsheet-lyme-borreliosis.pdf (accessed on 17 December 2022).
3. Marques, A.R.; Strle, F.; Wormser, G.P. Comparison of Lyme Disease in the United States and Europe. *Emerg. Infect. Dis.* **2021**, *27*, 2017–2024. https://doi.org/10.3201/eid2708.204763.
4. Földvári, G.; Široký, P.; Szekeres, S.; Majoros, G.; Sprong, H. *Dermacentor reticulatus*: A Vector on the Rise. *Parasites Vectors* **2016**, *9*, 314. https://doi.org/10.1186/s13071-016-1599-x.
5. Heglasová, I.; Rudenko, N.; Golovchenko, M.; Zubriková, D.; Miklisová, D.; Stanko, M. Ticks, Fleas and Rodent-Hosts Analyzed for the Presence of *Borrelia miyamotoi* in Slovakia: The First Record of *Borrelia miyamotoi* in a Haemaphysalis Inermis Tick. *Ticks Tick Borne Dis.* **2020**, *11*, 101456. https://doi.org/10.1016/j.ttbdis.2020.101456.
6. Del Cerro, A.; Oleaga, A.; Somoano, A.; Barandika, J.F.; García-Pérez, A.L.; Espí, A. Molecular Identification of Tick-Borne Pathogens (*Rickettsia* Spp., *Anaplasma phagocytophilum*, *Borrelia burgdorferi sensu lato*, *Coxiella burnetii* and Piroplasms) in Questing and Feeding Hard Ticks from North-Western Spain. *Ticks Tick Borne Dis.* **2022**, *13*, 101961. https://doi.org/10.1016/j.ttbdis.2022.101961.
7. Grover, A.K.; Singh, P.; Puri, S.; Jindal, S.; Choudhary, P.N. Dog Tick (*Rhipecephalus*) Causing Lyme Disease in an Adult Human. *J. Fam. Med. Prim. Care* **2022**, *11*, 4824–4826. https://doi.org/10.4103/jfmpc.jfmpc_33_22.
8. Ji, Z.; Jian, M.; Yue, P.; Cao, W.; Xu, X.; Zhang, Y.; Pan, Y.; Yang, J.; Chen, J.; Liu, M.; et al. Prevalence of *Borrelia Burgdorferi* in Ixodidae Tick around Asia: A Systematic Review and Meta-Analysis. *Pathogens* **2022**, *11*, 143. https://doi.org/10.3390/pathogens11020143.
9. Nagarajan, A.; Skufca, J.; Vyse, A.; Pilz, A.; Begier, E.; Riera-Montes, M.; Gessner, B.D.; Stark, J.H. The Landscape of Lyme Borreliosis Surveillance in Europe. *Vector Borne Zoonotic Dis.* **2023**, *23*, 142–155. https://doi.org/10.1089/vbz.2022.0067.
10. ECDC Comment: European Commission Updates Communicable Disease Surveillance List—Lyme Neuroborreliosis Now under EU/EEA Surveillance. Available online: https://www.ecdc.europa.eu/en/news-events/ecdc-comment-european-commission-updates-communicable-disease-surveillance-list-lyme (accessed on 17 December 2022).
11. Tick Maps. Available online: https://www.ecdc.europa.eu/en/disease-vectors/surveillance-and-disease-data/tick-maps (accessed on 25 May 2023).
12. Lyme Borreliosis in Europe. Available online: https://climate-adapt.eea.europa.eu/en/metadata/publications/lyme-borreliosis-in-europe (accessed on 17 December 2022).
13. Lyme Borreliosis in Europe: Influences of Climate and Climate Change, Epidemiology, Ecology and Adaptation Measures. Available online: https://www.who.int/publications/i/item/9789289022910 (accessed on 25 May 2023).
14. Voyiatzaki, C.; Papailia, S.I.; Venetikou, M.S.; Pouris, J.; Tsoumani, M.E.; Papageorgiou, E.G. Climate Changes Exacerbate the Spread of *Ixodes ricinus* and the Occurrence of Lyme Borreliosis and Tick-Borne Encephalitis in Europe—How Climate Models Are Used as a Risk Assessment Approach for Tick-Borne Diseases. *Int. J. Environ. Res. Public Health* **2022**, *19*, 6516. https://doi.org/10.3390/ijerph19116516.
15. Wongnak, P.; Bord, S.; Jacquot, M.; Agoulon, A.; Beugnet, F.; Bournez, L.; Cèbe, N.; Chevalier, A.; Cosson, J.-F.; Dambrine, N.; et al. Meteorological and Climatic Variables Predict the Phenology of *Ixodes ricinus* Nymph Activity in France, Accounting for Habitat Heterogeneity. *Sci. Rep.* **2022**, *12*, 7833. https://doi.org/10.1038/s41598-022-11479-z.
16. Moher, D.; Shamseer, L.; Clarke, M.; Ghersi, D.; Liberati, A.; Petticrew, M.; Shekelle, P.; Stewart, L.A.; PRISMA-P Group Preferred Reporting Items for Systematic Review and Meta-Analysis Protocols (PRISMA-P) 2015 Statement. *Syst. Rev.* **2015**, *4*, 1. https://doi.org/10.1186/2046-4053-4-1.
17. Giesen, C.; Roche, J.; Redondo-Bravo, L.; Ruiz-Huerta, C.; Gomez-Barroso, D.; Benito, A.; Herrador, Z. The Impact of Climate Change on Mosquito-Borne Diseases in Africa. *Pathog. Glob. Health* **2020**, *114*, 287–301. https://doi.org/10.1080/20477724.2020.1783865.
18. Brugueras, S.; Fernández-Martínez, B.; Martínez-de la Puente, J.; Figuerola, J.; Porro, T.M.; Rius, C.; Larrauri, A.; Gómez-Barroso, D. Environmental Drivers, Climate Change and Emergent Diseases Transmitted by Mosquitoes and Their Vectors in Southern Europe: A Systematic Review. *Environ. Res.* **2020**, *191*, 110038. https://doi.org/10.1016/j.envres.2020.110038.
19. Ottawa Hospital Research Institute. Available online: https://www.ohri.ca/programs/clinical_epidemiology/oxford.asp (accessed on 22 May 2023).
20. Linard, C.; Lamarque, P.; Heyman, P.; Ducoffre, G.; Luyasu, V.; Tersago, K.; Vanwambeke, S.O.; Lambin, E.F. Determinants of the Geographic Distribution of Puumala Virus and Lyme Borreliosis Infections in Belgium. *Int. J. Health Geogr.* **2007**, *6*, 15. https://doi.org/10.1186/1476-072X-6-15.
21. Heylen, D.; Lasters, R.; Adriaensen, F.; Fonville, M.; Sprong, H.; Matthysen, E. Ticks and Tick-Borne Diseases in the City: Role of Landscape Connectivity and Green Space Characteristics in a Metropolitan Area. *Sci. Total Environ.* **2019**, *670*, 941–949. https://doi.org/10.1016/j.scitotenv.2019.03.235.

22. Keukeleire, M.D.; Robert, A.; Kabamba, B.; Dion, E.; Luyasu, V.; Vanwambeke, S.O. Individual and Environmental Factors Associated with the Seroprevalence of *Borrelia burgdorferi* in Belgian Farmers and Veterinarians. *Infect. Ecol. Epidemiol.* **2016**, *6*, 32793. https://doi.org/10.3402/iee.v6.32793.
23. Barrios, J.M.; Verstraeten, W.W.; Maes, P.; Aerts, J.M.; Farifteh, J.; Coppin, P. Seasonal Vegetation Variables and Their Impact on the Spatio-Temporal Patterns of Nephropathia Epidemica and Lyme Borreliosis in Belgium. *Appl. Geogr.* **2013**, *45*, 230–240. https://doi.org/10.1016/j.apgeog.2013.09.019.
24. Tack, W.; Madder, M.; Baeten, L.; Vanhellemont, M.; Gruwez, R.; Verheyen, K. Local Habitat and Landscape Affect *Ixodes ricinus* Tick Abundances in Forests on Poor, Sandy Soils. *For. Ecol. Manag.* **2012**, *265*, 30–36. https://doi.org/10.1016/j.foreco.2011.10.028.
25. Barrios, J.; Verstraeten, W.; Maes, P.; Aerts, J.-M.; Farifteh, J.; Coppin, P. Using the Gravity Model to Estimate the Spatial Spread of Vector-Borne Diseases. *IJERPH* **2012**, *9*, 4346–4364. https://doi.org/10.3390/ijerph9124346.
26. Barrios, J.M.; Verstraeten, W.W.; Maes, P.; Clement, J.; Aerts, J.M.; Farifteh, J.; Lagrou, K.; Van Ranst, M.; Coppin, P. Remotely Sensed Vegetation Moisture as Explanatory Variable of Lyme Borreliosis Incidence. *Int. J. Appl. Earth Obs. Geoinf.* **2012**, *18*, 1–12. https://doi.org/10.1016/j.jag.2012.01.023.
27. Heylen, D.; Adriaensen, F.; Van Dongen, S.; Sprong, H.; Matthysen, E. Ecological Factors That Determine *Ixodes ricinus* Tick Burdens in the Great Tit (Parus Major), an Avian Reservoir of *Borrelia burgdorferi* s.l. *Int. J. Parasitol.* **2013**, *43*, 603–611. https://doi.org/10.1016/j.ijpara.2013.02.007.
28. Hönig, V.; Svec, P.; Halas, P.; Vavruskova, Z.; Tykalova, H.; Kilian, P.; Vetiskova, V.; Dornakova, V.; Sterbova, J.; Simonova, Z.; et al. Ticks and Tick-Borne Pathogens in South Bohemia (Czech Republic)—Spatial Variability in *Ixodes ricinus* Abundance, *Borrelia burgdorferi* and Tick-Borne Encephalitis Virus Prevalence. *Ticks Tick-Borne Dis.* **2015**, *6*, 559–567. https://doi.org/10.1016/j.ttbdis.2015.04.010.
29. Daniel, M.; Materna, J.; Hönig, V.; Metelka, L.; Danielová, V.; Harčarik, J.; Kliegrová, S.; Grubhoffer, L. Vertical Distribution of the Tick *Ixodes ricinus* and Tick-Borne Pathogens in the Northern Moravian Mountains Correlated with Climate Warming (Jeseníky Mts., Czech Republic). *Cent. Eur. J. Public Health* **2009**, *17*, 139–145. https://doi.org/10.21101/cejph.a3550.
30. Daniel, M.; Kříž, B.; Valter, J.; Kott, I.; Danielová, V. The Influence of Meteorological Conditions of the Preceding Winter on the Incidences of Tick-Borne Encephalitis and Lyme Borreliosis in the Czech Republic. *Int. J. Med. Microbiol.* **2008**, *298*, 60–67. https://doi.org/10.1016/j.ijmm.2008.05.001.
31. Hubálek, Z. North Atlantic Weather Oscillation and Human Infectious Diseases in the Czech Republic, 1951–2003. *Eur. J. Epidemiol.* **2005**, *20*, 263–270. https://doi.org/10.1007/s10654-004-6518-3.
32. Daniel, M.; Malý, M.; Danielová, V.; Kříž, B.; Nuttall, P. Abiotic Predictors and Annual Seasonal Dynamics of *Ixodes ricinus*, the Major Disease Vector of Central Europe. *Parasites Vectors* **2015**, *8*, 478. https://doi.org/10.1186/s13071-015-1092-y.
33. Hubálek, Z.; Halouzka, J.; Juricová, Z. Host-Seeking Activity of Ixodid Ticks in Relation to Weather Variables. *J. Vector Ecol.* **2003**, *28*, 159–165.
34. Tkadlec, E.; Václavík, T.; Široký, P. Rodent Host Abundance and Climate Variability as Predictors of Tickborne Disease Risk 1 Year in Advance. *Emerg. Infect. Dis.* **2019**, *25*, 1738–1741. https://doi.org/10.3201/eid2509.190684.
35. Jensen, P.M.; Jespersen, J.B. Five Decades of Tick–Man Interaction in Denmark—An Analysis. *Exp. Appl. Acarol.* **2005**, *35*, 131–146. https://doi.org/10.1007/s10493-004-1991-7.
36. Jensen, P.M.; Hansen, H. Spatial Risk Assessment for Lyme Borreliosis in Denmark. *Scand. J. Infect. Dis.* **2000**, *32*, 545–550. https://doi.org/10.1080/003655400458857.
37. Jensen, P.M.; Frand, F. Temporal Risk Assessment for Lyme Borreliosis in Denmark. *Scand. J. Infect. Dis.* **2000**, *32*, 539–544. https://doi.org/10.1080/003655400458848.
38. Kjær, L.J.; Soleng, A.; Edgar, K.S.; Lindstedt, H.E.H.; Paulsen, K.M.; Andreassen, Å.K.; Korslund, L.; Kjelland, V.; Slettan, A.; Stuen, S.; et al. Predicting and Mapping Human Risk of Exposure to *Ixodes ricinus* Nymphs Using Climatic and Environmental Data, Denmark, Norway and Sweden, 2016. *Eurosurveillance* **2019**, *24*, 1800101. https://doi.org/10.2807/1560-7917.ES.2019.24.9.1800101.
39. Kjær, L.J.; Soleng, A.; Edgar, K.S.; Lindstedt, H.E.H.; Paulsen, K.M.; Andreassen, Å.K.; Korslund, L.; Kjelland, V.; Slettan, A.; Stuen, S.; et al. Predicting the Spatial Abundance of *Ixodes ricinus* Ticks in Southern Scandinavia Using Environmental and Climatic Data. *Sci. Rep.* **2019**, *9*, 18144. https://doi.org/10.1038/s41598-019-54496-1.
40. Porretta, D.; Mastrantonio, V.; Amendolia, S.; Gaiarsa, S.; Epis, S.; Genchi, C.; Bandi, C.; Otranto, D.; Urbanelli, S. Effects of Global Changes on the Climatic Niche of the Tick *Ixodes ricinus* Inferred by Species Distribution Modelling. *Parasites Vectors* **2013**, *6*, 271. https://doi.org/10.1186/1756-3305-6-271.
41. Li, S.; Heyman, P.; Cochez, C.; Simons, L.; Vanwambeke, S.O. A Multi-Level Analysis of the Relationship between Environmental Factors and Questing *Ixodes ricinus* Dynamics in Belgium. *Parasites Vectors* **2012**, *5*, 149. https://doi.org/10.1186/1756-3305-5-149.

42. Li, S.; Gilbert, L.; Vanwambeke, S.O.; Yu, J.; Purse, B.V.; Harrison, P.A. Lyme Disease Risks in Europe under Multiple Uncertain Drivers of Change. *Environ. Health Perspect.* **2019**, *127*, 67010. https://doi.org/10.1289/EHP4615.
43. Fernández-Ruiz, N.; Estrada-Peña, A. Could Climate Trends Disrupt the Contact Rates between *Ixodes ricinus* (Acari, Ixodidae) and the Reservoirs of *Borrelia burgdorferi* s.l. *PLoS ONE* **2020**, *15*, e0233771. https://doi.org/10.1371/journal.pone.0233771.
44. Uusitalo, R.; Siljander, M.; Lindén, A.; Sormunen, J.J.; Aalto, J.; Hendrickx, G.; Kallio, E.; Vajda, A.; Gregow, H.; Henttonen, H.; et al. Predicting Habitat Suitability for *Ixodes ricinus* and *Ixodes persulcatus* Ticks in Finland. *Parasites Vectors* **2022**, *15*, 310. https://doi.org/10.1186/s13071-022-05410-8.
45. Mariet, A.-S.; Retel, O.; Avocat, H.; Serre, A.; Schapman, L.; Schmitt, M.; Charron, M.; Monnet, E. Estimated Incidence of Erythema Migrans in Five Regions of France and Ecological Correlations with Environmental Characteristics. *Vector-Borne Zoonotic Dis.* **2013**, *13*, 666–673. https://doi.org/10.1089/vbz.2012.1199.
46. Vassallo, M.; Paul, R.E.L.; Pérez-Eid, C. Temporal distribution of the annual nymphal stock of *Ixodes ricinus* ticks. *Exp. Appl. Acarol.* **2000**, *24*, 941–949. https://doi.org/10.1023/A:1010669003887.
47. Goldstein, V.; Boulanger, N.; Schwartz, D.; George, J.-C.; Ertlen, D.; Zilliox, L.; Schaeffer, M.; Jaulhac, B. Factors Responsible for *Ixodes ricinus* Nymph Abundance: Are Soil Features Indicators of Tick Abundance in a French Region Where Lyme Borreliosis Is Endemic? *Ticks Tick-Borne Dis.* **2018**, *9*, 938–944. https://doi.org/10.1016/j.ttbdis.2018.03.013.
48. Vourc'h, G.; Abrial, D.; Bord, S.; Jacquot, M.; Masséglia, S.; Poux, V.; Pisanu, B.; Bailly, X.; Chapuis, J.-L. Mapping Human Risk of Infection with *Borrelia burgdorferi sensu lato*, the Agent of Lyme Borreliosis, in a Periurban Forest in France. *Ticks Tick-Borne Dis.* **2016**, *7*, 644–652. https://doi.org/10.1016/j.ttbdis.2016.02.008.
49. Paul, R.E.L.; Cote, M.; Le Naour, E.; Bonnet, S.I. Environmental Factors Influencing Tick Densities over Seven Years in a French Suburban Forest. *Parasites Vectors* **2016**, *9*, 309. https://doi.org/10.1186/s13071-016-1591-5.
50. Halos, L.; Bord, S.; Cotté, V.; Gasqui, P.; Abrial, D.; Barnouin, J.; Boulouis, H.-J.; Vayssier-Taussat, M.; Vourc'h, G. Ecological Factors Characterizing the Prevalence of Bacterial Tick-Borne Pathogens in *Ixodes ricinus* Ticks in Pastures and Woodlands. *Appl. Environ. Microbiol.* **2010**, *76*, 4413–4420. https://doi.org/10.1128/AEM.00610-10.
51. Boyard, C.; Barnouin, J.; Gasqui, P.; Vourc'H, G. Local Environmental Factors Characterizing *Ixodes ricinus* Nymph Abundance in Grazed Permanent Pastures for Cattle. *Parasitology* **2007**, *134*, 987–994. https://doi.org/10.1017/S0031182007002351.
52. Bourdin, A.; Bord, S.; Durand, J.; Galon, C.; Moutailler, S.; Scherer-Lorenzen, M.; Jactel, H. Forest Diversity Reduces the Prevalence of Pathogens Transmitted by the Tick *Ixodes ricinus*. *Front. Ecol. Evol.* **2022**, *10*, 891908. https://doi.org/10.3389/fevo.2022.891908.
53. Perez, G.; Bastian, S.; Agoulon, A.; Bouju, A.; Durand, A.; Faille, F.; Lebert, I.; Rantier, Y.; Plantard, O.; Butet, A. Effect of Landscape Features on the Relationship between *Ixodes ricinus* Ticks and Their Small Mammal Hosts. *Parasites Vectors* **2016**, *9*, 20. https://doi.org/10.1186/s13071-016-1296-9.
54. Ehrmann, S.; Liira, J.; Gärtner, S.; Hansen, K.; Brunet, J.; Cousins, S.A.O.; Deconchat, M.; Decocq, G.; De Frenne, P.; De Smedt, P.; et al. Environmental Drivers of *Ixodes ricinus* Abundance in Forest Fragments of Rural European Landscapes. *BMC Ecol.* **2017**, *17*, 31. https://doi.org/10.1186/s12898-017-0141-0.
55. Ehrmann, S.; Ruyts, S.C.; Scherer-Lorenzen, M.; Bauhus, J.; Brunet, J.; Cousins, S.A.O.; Deconchat, M.; Decocq, G.; De Frenne, P.; De Smedt, P.; et al. Habitat Properties Are Key Drivers of *Borrelia burgdorferi* (s.l.) Prevalence in *Ixodes ricinus* Populations of Deciduous Forest Fragments. *Parasites Vectors* **2018**, *11*, 23. https://doi.org/10.1186/s13071-017-2590-x.
56. Brugger, K.; Walter, M.; Chitimia-Dobler, L.; Dobler, G.; Rubel, F. Forecasting next Season's *Ixodes ricinus* Nymphal Density: The Example of Southern Germany 2018. *Exp. Appl. Acarol.* **2018**, *75*, 281–288. https://doi.org/10.1007/s10493-018-0267-6.
57. Nolzen, H.; Brugger, K.; Reichold, A.; Brock, J.; Lange, M.; Thulke, H.-H. Model-Based Extrapolation of Ecological Systems under Future Climate Scenarios: The Example of *Ixodes ricinus* Ticks. *PLoS ONE* **2022**, *17*, e0267196. https://doi.org/10.1371/journal.pone.0267196.
58. Răileanu, C.; Silaghi, C.; Fingerle, V.; Margos, G.; Thiel, C.; Pfister, K.; Overzier, E. *Borrelia burgdorferi* Sensu Lato in Questing and Engorged Ticks from Different Habitat Types in Southern Germany. *Microorganisms* **2021**, *9*, 1266. https://doi.org/10.3390/microorganisms9061266.
59. Kohn, M.; Krücken, J.; McKay-Demeler, J.; Pachnicke, S.; Krieger, K.; von Samson-Himmelstjerna, G. *Dermacentor reticulatus* in Berlin/Brandenburg (Germany): Activity Patterns and Associated Pathogens. *Ticks Tick-Borne Dis.* **2019**, *10*, 191–206. https://doi.org/10.1016/j.ttbdis.2018.10.003.
60. Brugger, K.; Walter, M.; Chitimia-Dobler, L.; Dobler, G.; Rubel, F. Seasonal Cycles of the TBE and Lyme Borreliosis Vector *Ixodes ricinus* Modelled with Time-Lagged and Interval-Averaged Predictors. *Exp. Appl. Acarol.* **2017**, *73*, 439–450. https://doi.org/10.1007/s10493-017-0197-8.

61. Boehnke, D.; Brugger, K.; Pfäffle, M.; Sebastian, P.; Norra, S.; Petney, T.; Oehme, R.; Littwin, N.; Lebl, K.; Raith, J.; et al. Estimating *Ixodes ricinus* Densities on the Landscape Scale. *Int. J. Health Geogr.* **2015**, *14*, 23. https://doi.org/10.1186/s12942-015-0015-7.
62. Schwarz, A.; Maier, W.A.; Kistemann, T.; Kampen, H. Analysis of the Distribution of the Tick *Ixodes ricinus* L. (Acari: Ixodidae) in a Nature Reserve of Western Germany Using Geographic Information Systems. *Int. J. Hyg. Environ. Health* **2009**, *212*, 87–96. https://doi.org/10.1016/j.ijheh.2007.12.001.
63. Vollack, K.; Sodoudi, S.; Névir, P.; Müller, K.; Richter, D. Influence of Meteorological Parameters during the Preceding Fall and Winter on the Questing Activity of Nymphal *Ixodes ricinus* Ticks. *Int. J. Biometeorol.* **2017**, *61*, 1787–1795. https://doi.org/10.1007/s00484-017-1362-9.
64. Schulz, M.; Mahling, M.; Pfister, K. Abundance and Seasonal Activity of Questing *Ixodes ricinus* Ticks in Their Natural Habitats in Southern Germany in 2011. *J. Vector Ecol.* **2014**, *39*, 56–65. https://doi.org/10.1111/j.1948-7134.2014.12070.x.
65. Gethmann, J.; Hoffmann, B.; Kasbohm, E.; Süss, J.; Habedank, B.; Conraths, F.J.; Beer, M.; Klaus, C. Research Paper on Abiotic Factors and Their Influence on *Ixodes ricinus* Activity—Observations over a Two-Year Period at Several Tick Collection Sites in Germany. *Parasitol. Res.* **2020**, *119*, 1455–1466. https://doi.org/10.1007/s00436-020-06666-8.
66. Lauterbach, R.; Wells, K.; O'Hara, R.B.; Kalko, E.K.V.; Renner, S.C. Variable Strength of Forest Stand Attributes and Weather Conditions on the Questing Activity of *Ixodes ricinus* Ticks over Years in Managed Forests. *PLoS ONE* **2013**, *8*, e55365. https://doi.org/10.1371/journal.pone.0055365.
67. Hauck, D.; Springer, A.; Chitimia-Dobler, L.; Strube, C. Two-Year Monitoring of Tick Abundance and Influencing Factors in an Urban Area (City of Hanover, Germany). *Ticks Tick-Borne Dis.* **2020**, *11*, 101464. https://doi.org/10.1016/j.ttbdis.2020.101464.
68. Trájer, A.; Bobvos, J.; Páldy, A.; Krisztalovics, K. Association between Incidence of Lyme Disease and Spring-Early Summer Season Temperature Changes in Hungary—1998–2010. *Ann. Agric. Environ. Med.* **2013**, *20*, 245–251.
69. Trájer, A.; Bede-Fazekas, Á.; Hufnagel, L.; Bobvos, J.; Páldy, A. The Paradox of the Binomial *Ixodes ricinus* Activity and the Observed Unimodal Lyme Borreliosis Season in Hungary. *Int. J. Environ. Health Res.* **2014**, *24*, 226–245. https://doi.org/10.1080/09603123.2013.807329.
70. Hornok, S.; Mulvihill, M.; Szőke, K.; Gönczi, E.; Sulyok, K.M.; Gyuranecz, M.; Hofmann-Lehmann, R. Impact of a Freeway on the Dispersal of Ticks and *Ixodes ricinus*-Borne Pathogens: Forested Resting Areas May Become Lyme Disease Hotspots. *Acta Vet. Hung.* **2017**, *65*, 242–252. https://doi.org/10.1556/004.2017.024.
71. Garcia-Vozmediano, A.; Krawczyk, A.I.; Sprong, H.; Rossi, L.; Ramassa, E.; Tomassone, L. Ticks Climb the Mountains: Ixodid Tick Infestation and Infection by Tick-Borne Pathogens in the Western Alps. *Ticks Tick-Borne Dis.* **2020**, *11*, 101489. https://doi.org/10.1016/j.ttbdis.2020.101489.
72. Rosà, R.; Pugliese, A.; Ghosh, M.; Perkins, S.E.; Rizzoli, A. Temporal Variation of *Ixodes ricinus* Intensity on the Rodent Host *Apodemus flavicollis* in Relation to Local Climate and Host Dynamics. *Vector-Borne Zoonotic Dis.* **2007**, *7*, 285–295. https://doi.org/10.1089/vbz.2006.0607.
73. Rizzoli, A.; Merler, S.; Furlanello, C.; Genchi, C. Geographical Information Systems and Bootstrap Aggregation (Bagging) of Tree-Based Classifiers for Lyme Disease Risk Prediction in Trentino, Italian Alps. *J. Med. Entomol.* **2002**, *39*, 485–492. https://doi.org/10.1603/0022-2585-39.3.485.
74. Zanzani, S.A.; Rimoldi, S.G.; Manfredi, M.; Grande, R.; Gazzonis, A.L.; Merli, S.; Olivieri, E.; Giacomet, V.; Antinori, S.; Cislaghi, G.; et al. Lyme Borreliosis Incidence in Lombardy, Italy (2000–2015): Spatiotemporal Analysis and Environmental Risk Factors. *Ticks Tick-Borne Dis.* **2019**, *10*, 101257. https://doi.org/10.1016/j.ttbdis.2019.07.001.
75. Altobelli, A.; Boemo, B.; Mignozzi, K.; Bandi, M.; Floris, R.; Menardi, G.; Cinco, M. Spatial Lyme Borreliosis Risk Assessment in North-Eastern Italy. *Int. J. Med. Microbiol.* **2008**, *298*, 125–128. https://doi.org/10.1016/j.ijmm.2008.05.005.
76. Bisanzio, D.; Amore, G.; Ragagli, C.; Tomassone, L.; Bertolotti, L.; Mannelli, A. Temporal Variations in the Usefulness of Normalized Difference Vegetation Index as a Predictor for *Ixodes ricinus* (Acari: Ixodidae) in a *Borrelia lusitaniae* Focus in Tuscany, Central Italy. *J. Med. Entomol.* **2008**, *45*, 547–555. https://doi.org/10.1603/0022-2585(2008)45[547:TVITUO]2.0.CO;2.
77. Tagliapietra, V.; Rosà, R.; Arnoldi, D.; Cagnacci, F.; Capelli, G.; Montarsi, F.; Hauffe, H.C.; Rizzoli, A. Saturation Deficit and Deer Density Affect Questing Activity and Local Abundance of *Ixodes ricinus* (Acari, Ixodidae) in Italy. *Vet. Parasitol.* **2011**, *183*, 114–124. https://doi.org/10.1016/j.vetpar.2011.07.022.
78. Rosà, R.; Andreo, V.; Tagliapietra, V.; Baráková, I.; Arnoldi, D.; Hauffe, H.; Manica, M.; Rosso, F.; Blaňarová, L.; Bona, M.; et al. Effect of Climate and Land Use on the Spatio-Temporal Variability of Tick-Borne Bacteria in Europe. *IJERPH* **2018**, *15*, 732. https://doi.org/10.3390/ijerph15040732.
79. Garcia-Martí, I.; Zurita-Milla, R.; Swart, A.; van den Wijngaard, K.C.; van Vliet, A.J.H.; Bennema, S.; Harms, M. Identifying Environmental and Human Factors Associated With Tick Bites Using Volunteered Reports and Frequent Pattern Mining. *Trans. GIS* **2017**, *21*, 277–299. https://doi.org/10.1111/tgis.12211.

80. Swart, A.; Ibañez-Justicia, A.; Buijs, J.; van Wieren, S.E.; Hofmeester, T.R.; Sprong, H.; Takumi, K. Predicting Tick Presence by Environmental Risk Mapping. *Front. Public Health* **2014**, *2*, 238. https://doi.org/10.3389/fpubh.2014.00238.
81. Qviller, L.; Grøva, L.; Viljugrein, H.; Klingen, I.; Mysterud, A. Temporal Pattern of Questing Tick *Ixodes ricinus* Density at Differing Elevations in the Coastal Region of Western Norway. *Parasites Vectors* **2014**, *7*, 179. https://doi.org/10.1186/1756-3305-7-179.
82. Kiewra, D.; Szymanowski, M.; Zalewska, G.; Dobracka, B.; Dobracki, W.; Klakočar, J.; Czułowska, A.; Plewa-Tutaj, K. Seroprevalence of *Borrelia burgdorferi* in Forest Workers from Inspectorates with Different Forest Types in Lower Silesia, SW Poland: Preliminary Study. *Int. J. Environ. Health Res.* **2018**, *28*, 502–510. https://doi.org/10.1080/09603123.2018.1489954.
83. Buczek, A.; Ciura, D.; Bartosik, K.; Zając, Z.; Kulisz, J. Threat of Attacks of *Ixodes ricinus* Ticks (Ixodida: Ixodidae) and Lyme Borreliosis within Urban Heat Islands in South-Western Poland. *Parasites Vectors* **2014**, *7*, 562. https://doi.org/10.1186/s13071-014-0562-y.
84. Dyczko, D.; Kiewra, D.; Kolanek, A.; Błażej, P. The Influence of Local Environmental Factors in Southwestern Poland on the Abundance of *Ixodes ricinus* and Prevalence of Infection with *Borrelia burgdorferi* s.l. and *B. miyamotoi*. *Parasitol. Res.* **2022**, *121*, 1575–1585. https://doi.org/10.1007/s00436-022-07493-9.
85. Kiewra, D.; Kryza, M.; Szymanowski, M. Influence of Selected Meteorological Variables on the Questing Activity of *Ixodes ricinus* Ticks in Lower Silesia, SW Poland. *J. Vector Ecol.* **2014**, *39*, 138–145. https://doi.org/10.1111/j.1948-7134.2014.12080.x.
86. Domșa, C. Modeling the Distribution of *Ixodes ricinus* in Romania. *North-West. J. Zool.* **2018**, *14*, 25–29.
87. Pangrácová, L.; Derdáková, M.; Pekárik, L.; Hviščová, I.; Víchová, B.; Stanko, M.; Hlavatá, H.; Peťko, B. *Ixodes ricinus* Abundance and Its Infection with the Tick-Borne Pathogens in Urban and Suburban Areas of Eastern Slovakia. *Parasites Vectors* **2013**, *6*, 238. https://doi.org/10.1186/1756-3305-6-238.
88. Kazimírová, M.; Hamšíková, Z.; Kocianová, E.; Marini, G.; Mojšová, M.; Mahríková, L.; Berthová, L.; Slovák, M.; Rosá, R. Relative Density of Host-Seeking Ticks in Different Habitat Types of South-Western Slovakia. *Exp. Appl. Acarol.* **2016**, *69*, 205–224. https://doi.org/10.1007/s10493-016-0025-6.
89. Donša, D.; Grujić, V.J.; Pipenbaher, N.; Ivajnšič, D. The Lyme Borreliosis Spatial Footprint in the 21st Century: A Key Study of Slovenia. *Int. J. Environ. Res. Public Health* **2021**, *18*, 12061. https://doi.org/10.3390/ijerph182212061.
90. Knap, N.; Durmiši, E.; Saksida, A.; Korva, M.; Petrovec, M.; Avšič-Županc, T. Influence of Climatic Factors on Dynamics of Questing *Ixodes ricinus* Ticks in Slovenia. *Vet. Parasitol.* **2009**, *164*, 275–281. https://doi.org/10.1016/j.vetpar.2009.06.001.
91. Ruiz-Fons, F.; Fernández-de-Mera, I.G.; Acevedo, P.; Gortázar, C.; de la Fuente, J. Factors Driving the Abundance of *Ixodes ricinus* Ticks and the Prevalence of Zoonotic *I. ricinus*-Borne Pathogens in Natural Foci. *Appl. Environ. Microbiol.* **2012**, *78*, 2669–2676. https://doi.org/10.1128/AEM.06564-11.
92. Estrada-Peña, A. Distribution, Abundance, and Habitat Preferences of *Ixodes ricinus* (Acari: Ixodidae) in Northern Spain. *J. Med. Entomol.* **2001**, *38*, 361–370. https://doi.org/10.1603/0022-2585-38.3.361.
93. Alonso-Carné, J.; García-Martín, A.; Estrada-Peña, A. Modelling the Phenological Relationships of Questing Immature *Ixodes ricinus* (Ixodidae) Using Temperature and NDVI Data. *Zoonoses Public Health* **2016**, *63*, 40–52. https://doi.org/10.1111/zph.12203.
94. Barandika, J.F.; Berriatua, E.; Barral, M.; Juste, R.A.; Anda, P.; Garcia-Perez, A.L. Risk Factors Associated with Ixodid Tick Species Distributions in the Basque Region in Spain. *Med. Vet. Entomol.* **2006**, *20*, 177–188. https://doi.org/10.1111/j.1365-2915.2006.00619.x.
95. Bennet, L.; Halling, A.; Berglund, J. Increased Incidence of Lyme Borreliosis in Southern Sweden Following Mild Winters and during Warm, Humid Summers. *Eur. J. Clin. Microbiol. Infect. Dis.* **2006**, *25*, 426–432. https://doi.org/10.1007/s10096-006-0167-2.
96. Jaenson, T.G.T.; Eisen, L.; Comstedt, P.; Mejlon, H.A.; Lindgren, E.; Bergström, S.; Olsen, B. Risk Indicators for the Tick *Ixodes ricinus* and *Borrelia burgdorferi* Sensu Lato in Sweden. *Med. Vet. Entomol.* **2009**, *23*, 226–237. https://doi.org/10.1111/j.1365-2915.2009.00813.x.
97. Jaenson, T.G.T.; Lindgren, E. The Range of *Ixodes ricinus* and the Risk of Contracting Lyme Borreliosis Will Increase Northwards When the Vegetation Period Becomes Longer. *Ticks Tick-Borne Dis.* **2011**, *2*, 44–49. https://doi.org/10.1016/j.ttbdis.2010.10.006.
98. Keith, K.; Årestedt, K.; Tjernberg, I. The Relationship between the Laboratory Diagnosis of Lyme Neuroborreliosis and Climate Factors in Kalmar County Sweden—An Overview between 2008 and 2019. *Eur. J. Clin. Microbiol. Infect. Dis.* **2022**, *41*, 253–261. https://doi.org/10.1007/s10096-021-04374-4.
99. Lindström, A.; Jaenson, T.G.T. Distribution of the Common Tick, *Ixodes ricinus* (Acari: Ixodidae), in Different Vegetation Types in Southern Sweden. *J. Med. Entomol.* **2003**, *40*, 375–378. https://doi.org/10.1603/0022-2585-40.4.375.

100. Markowicz, M.; Schötta, A.-M.; Höss, D.; Kundi, M.; Schray, C.; Stockinger, H.; Stanek, G. Infections with Tickborne Pathogens after Tick Bite, Austria, 2015–2018. *Emerg. Infect. Dis.* **2021**, *27*, 1048–1056. https://doi.org/10.3201/eid2704.203366.
101. Petrulionienė, A.; Radzišauskienė, D.; Ambrozaitis, A.; Čaplinskas, S.; Paulauskas, A.; Venalis, A. Epidemiology of Lyme Disease in a Highly Endemic European Zone. *Medicina* **2020**, *56*, 115. https://doi.org/10.3390/medicina56030115.
102. van den Wijngaard, C.C.; Hofhuis, A.; Simões, M.; Rood, E.; van Pelt, W.; Zeller, H.; Van Bortel, W. Surveillance Perspective on Lyme Borreliosis across the European Union and European Economic Area. *Eurosurveillance* **2017**, *22*, 30569. https://doi.org/10.2807/1560-7917.ES.2017.22.27.30569.
103. Main Climates of Europe—European Environment Agency. Available online: https://www.eea.europa.eu/data-and-maps/figures/climate (accessed on 20 June 2023).
104. Köhler, C.F.; Holding, M.L.; Sprong, H.; Jansen, P.A.; Esser, H.J. Biodiversity in the Lyme-Light: Ecological Restoration and Tick-Borne Diseases in Europe. *Trends Parasitol.* **2023**, *39*, 373–385. https://doi.org/10.1016/j.pt.2023.02.005.
105. Kilpatrick, A.M.; Dobson, A.D.M.; Levi, T.; Salkeld, D.J.; Swei, A.; Ginsberg, H.S.; Kjemtrup, A.; Padgett, K.A.; Jensen, P.M.; Fish, D.; et al. Lyme Disease Ecology in a Changing World: Consensus, Uncertainty and Critical Gaps for Improving Control. *Philos. Trans. R. Soc. B Biol. Sci.* **2017**, *372*, 20160117. https://doi.org/10.1098/rstb.2016.0117.
106. Tjernberg, I.; Lager, M.; Furset Jensen, G.; Eikeland, R.; Nyman, D.; Brudin, L.; Henningsson, A.J. Identification of Potential Biomarkers in Active Lyme Borreliosis. *PLoS ONE* **2023**, *18*, e0287586. https://doi.org/10.1371/journal.pone.0287586.
107. Nowakowski, J.; Schwartz, I.; Liveris, D.; Wang, G.; Rosenfeld, M.E.A.; Girao, G.; McKenna, D.; Nadelman, R.B.; Cavaliere, L.F.; Wormser, G.P.; et al. Laboratory Diagnostic Techniques for Patients with Early Lyme Disease Associated with Erythema Migrans: A Comparison of Different Techniques. *Clin. Infect. Dis.* **2001**, *33*, 2023–2027. https://doi.org/10.1086/324490.
108. Ferguson, T.; Curtis, R.; Fraysse, F.; Olds, T.; Dumuid, D.; Brown, W.; Esterman, A.; Maher, C. Weather Associations with Physical Activity, Sedentary Behaviour and Sleep Patterns of Australian Adults: A Longitudinal Study with Implications for Climate Change. *Int. J. Behav. Nutr. Phys. Act.* **2023**, *20*, 30. https://doi.org/10.1186/s12966-023-01414-4.
109. How Ticks Spread Disease. Available online: https://www.cdc.gov/ticks/life_cycle_and_hosts.html (accessed on 20 June 2023).
110. European Summer 2023: A Season of Contrasting Extremes—Copernicus. Available online: https://climate.copernicus.eu/european-summer-2023-season-contrasting-extremes (accessed on 7 March 2024).
111. Randolph, S.E. Evidence That Climate Change Has Caused "emergence" of Tick-Borne Diseases in Europe? *Int. J. Med. Microbiol. Suppl.* **2004**, *293*, 5–15. https://doi.org/10.1016/S1433-1128(04)80004-4.
112. Matuschka, F.R.; Heiler, M.; Eiffert, H.; Fischer, P.; Lotter, H.; Spielman, A. Diversionary Role of Hoofed Game in the Transmission of Lyme Disease Spirochetes. *Am. J. Trop. Med. Hyg.* **1993**, *48*, 693–699. https://doi.org/10.4269/ajtmh.1993.48.693.
113. Sala, V.; De Faveri, E. Epidemiology of Lyme Disease in Domestic and Wild Animals. *Open Dermatol. J.* **2016**, *10*, 15–26. https://doi.org/10.2174/1874372201610010015.
114. Dobson, A.D.M.; Randolph, S.E. Modelling the Effects of Recent Changes in Climate, Host Density and Acaricide Treatments on Population Dynamics of *Ixodes ricinus* in the UK: *Ixodes ricinus* Population Change Model. *J. Appl. Ecol.* **2011**, *48*, 1029–1037. https://doi.org/10.1111/j.1365-2664.2011.02004.x.
115. Bouchard, C.; Dumas, A.; Baron, G.; Bowser, N.; Leighton, P.A.; Lindsay, L.R.; Milord, F.; Ogden, N.H.; Aenishaenslin, C. Integrated Human Behavior and Tick Risk Maps to Prioritize Lyme Disease Interventions Using a "One Health" Approach. *Ticks Tick-Borne Dis.* **2023**, *14*, 102083. https://doi.org/10.1016/j.ttbdis.2022.102083.
116. AR6 Synthesis Report: Climate Change 2023. Available online: https://www.ipcc.ch/report/ar6/syr/ (accessed on 23 July 2023).
117. World Meteorological Organization. Available online: https://public.wmo.int/en (accessed on 14 July 2023).

Disclaimer/Publisher's Note: The statements, opinions and data contained in all publications are solely those of the individual author(s) and contributor(s) and not of MDPI and/or the editor(s). MDPI and/or the editor(s) disclaim responsibility for any injury to people or property resulting from any ideas, methods, instructions or products referred to in the content.
